# Reconstruction of regulatory and metabolic pathways in metal-reducing δ-proteobacteria

**DOI:** 10.1186/gb-2004-5-11-r90

**Published:** 2004-10-22

**Authors:** Dmitry A Rodionov, Inna Dubchak, Adam Arkin, Eric Alm, Mikhail S Gelfand

**Affiliations:** 1 Institute for Information Transmission Problems, Russian Academy of Sciences, Bolshoi Karetny per. 19, Moscow 127994, Russia; 2 Genomics Division, Lawrence Berkeley National Laboratory, Berkeley, CA 94720, USA; 3 Physical Biosciences Division, Lawrence Berkeley National Laboratory, Berkeley, CA 94720, USA; 4 Howard Hughes Medical Institute, Berkeley, CA 94720, USA; 5 University of California, Berkeley, CA 94720, USA; 6 State Scientific Center GosniiGenetika, 1st Dorozhny pr. 1, Moscow 117545, Russia

## Abstract

**Background:**

Relatively little is known about the genetic basis for the unique physiology of metal-reducing genera in the delta subgroup of the proteobacteria. The recent availability of complete finished or draft-quality genome sequences for seven representatives allowed us to investigate the genetic and regulatory factors in a number of key pathways involved in the biosynthesis of building blocks and cofactors, metal-ion homeostasis, stress response, and energy metabolism using a combination of regulatory sequence detection and analysis of genomic context.

**Results:**

In the genomes of δ-proteobacteria, we identified candidate binding sites for four regulators of known specificity (BirA, CooA, HrcA, sigma-32), four types of metabolite-binding riboswitches (*RFN*-, *THI*-, *B12*-elements and S-box), and new binding sites for the FUR, ModE, NikR, PerR, and ZUR transcription factors, as well as for the previously uncharacterized factors HcpR and LysX. After reconstruction of the corresponding metabolic pathways and regulatory interactions, we identified possible functions for a large number of previously uncharacterized genes covering a wide range of cellular functions.

**Conclusions:**

Phylogenetically diverse δ-proteobacteria appear to have homologous regulatory components. This study for the first time demonstrates the adaptability of the comparative genomic approach to *de novo *reconstruction of a regulatory network in a poorly studied taxonomic group of bacteria. Recent efforts in large-scale functional genomic characterization of *Desulfovibrio *species will provide a unique opportunity to test and expand our predictions.

## Abstract 2

A study of the genetic and regulatory factors in several biosynthesis, metal ion homeostasis, stress response, and energy metabolism pathways suggests that phylogenetically diverse δ-proteobacteria have homologous regulatory components.

## Background

The delta subdivision of proteobacteria is a very diverse group of Gram-negative microorganisms that include aerobic genera *Myxococcus *with complex developmental lifestyles and *Bdellovibrio*, which prey on other bacteria [1]. In this study, we focus on anaerobic metal-reducing δ-proteobacteria, seven representatives of which have been sequenced recently, providing an opportunity for comparative genomic analysis. Within this group, sulfate-reducing bacteria, including *Desulfovibrio *and *Desulfotalea *species, are metabolically and ecologically versatile prokaryotes often characterized by their ability to reduce sulfate to sulfide [2]. They can be found in aquatic habitats or waterlogged soils containing abundant organic material and sufficient levels of sulfate, and play a key role in the global sulfur and carbon cycles [1]. Industrial interest in sulfate reducers has focused on their role in corrosion of metal equipment and the souring of petroleum reservoirs, while their ability to reduce toxic heavy metals has drawn attention from researchers interested in exploiting this ability for bioremediation. Psychrophilic sulfate-reducing *Desulfotalea psychrophila *has been isolated from permanently cold arctic marine sediments [3]. In contrast to sulfate-reducing bacteria, the genera *Geobacter *and *Desulfuromonas *comprise dissimilative metal-reducing bacteria, which cannot reduce sulfate, but include representatives that require sulfur as a respiratory electron acceptor for oxidation of acetate to carbon dioxide [4]. These bacteria are an important component of the subsurface biota that oxidizes organic compounds, hydrogen or sulfur with the reduction of insoluble Fe(III) oxides [5], and have also been implicated in corrosion and toxic metal reduction.

Knowledge of transcriptional regulatory networks is essential for understanding cellular processes in bacteria. However, experimental data about regulation of gene expression in δ-proteobacteria are very limited. Different approaches could be used for identification of co-regulated genes (regulons). Transcriptional profiling using DNA microarrays allows one to compare the expression levels of thousands of genes in different experimental conditions, and is a valuable tool for dissecting bacterial adaptation to various environments. Computational approaches, on the other hand, provide an opportunity to describe regulons in poorly characterized genomes. Comparison of upstream sequences of genes can, in principle, identify co-regulated genes. From large-scale studies [6-9] and analyses of individual regulatory systems [10-14] it is clear that the comparative analysis of binding sites for transcriptional regulators is a powerful approach to the functional annotation of bacterial genomes. Additional techniques used in genome context analysis, such as chromosomal gene clustering, protein fusions and co-occurrence profiles, in combination with metabolic reconstruction, allow the inference of functional coupling between genes and the prediction of gene function [15].

Recent completion of finished and draft quality genome sequences for δ-proteobacteria provides an opportunity for comparative analysis of transcriptional regulation and metabolic pathways in these bacteria. The finished genomes include sulfate-reducing *Desulfovibrio vulgaris *[16], *D. desulfuricans G20*, and *Desulfotalea psychrophila*, as well as the sulfur-reducing *G. sulfurreducens *[17], while the *G. metallireducens *genome has been completed to draft quality. A mixture of *Desulfuromonas acetoxidans *and *Desulfuromonas palmitatis *has been sequenced, resulting in a large number of small scaffolds, the identity of which (*acetoxidans *or *palmitatis*) has not been determined, and we refer to this sequence set simply as *Desulfuromonas*. Though draft-quality sequence can make it difficult to assert with confidence the absence of any particular gene, we have included these genomes in our study because they do provide insight as to the presence or absence of entire pathways, they can be compared to the related finished genome of *G. sulfurreducens*, and because complete genome sequence is not necessary for the methodology we use to detect regulatory sequences.

In this comprehensive study, we identify a large number of regulatory elements in these δ-proteobacteria. Some of the corresponding regulons are highly conserved among various bacteria (for example, riboswitches, BirA, CIRCE), whereas others are specific only for δ-proteobacteria. We also present the reconstruction of a number of biosynthetic pathways and systems for metal-ion homeostasis and stress response in these bacteria. The most important result of this study is identification of a novel regulon involved in sulfate reduction and energy metabolism in sulfate-reducing bacteria, which is most probably controlled by a regulator from the CRP/FNR family.

## Results

The results are organized under four main headings for convenience. In the first, we analyze a number of specific regulons for biosynthesis of various amino acids and cofactors in δ-proteobacteria. Most of them are controlled by RNA regulatory elements, or riboswitches, that are highly conserved across bacteria [18]. In the next section we describe several regulons for the uptake and homeostasis of transition metal ions that are necessary for growth. These regulons operate by transcription factors that are homologous to factors in *Escherichia coli*, but are predicted to recognize entirely different DNA signals. We then describe two stress-response regulons: heat-shock regulons (σ^32 ^and HrcA/CIRCE), which operate by regulatory elements conserved in diverse bacteria, and newly identified peroxide stress response regulons that are quite diverse and conserved only in closely related species. Finally, we present a completely new global regulon in metal-reducing δ-proteobacteria, which includes various genes involved in energy metabolism and sulfate reduction.

### Biosynthesis and transport of vitamins and amino acids

#### Biotin

Biotin (vitamin H) is an essential cofactor for numerous biotin-dependent carboxylases in a variety of microorganisms [19]. The strict control of biotin biosynthesis is mediated by the bifunctional BirA protein, which acts both as a biotin-protein ligase and a transcriptional repressor of the biotin operon. The consensus binding signal of BirA is a palindromic sequence TTGTAAACC-[N_14/15_]-GGTTTACAA [20]. Consistent with the presence of the biotin repressor BirA, all bacteria in this study have one or two candidate BirA-binding sites per genome, depending on the operon organization of the biotin genes (Table 1). In the *Desulfovibrio *species, the predicted BirA site is located between the divergently transcribed biotin operon and the *birA *gene. In other genomes, candidate binding sites for BirA precede one or two separate biotin biosynthetic loci, whereas the *birA *gene stands apart and is not regulated.

**Figure 1:**
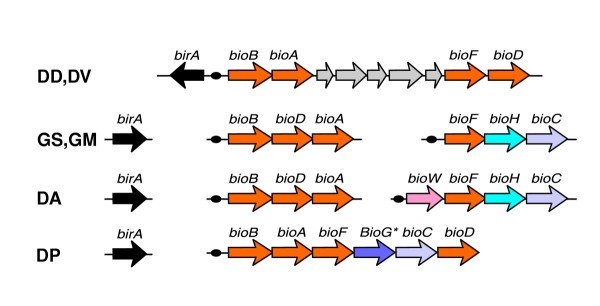
Genomic organization of the biotin biosynthetic genes and regulatory elements. DV (*Desulfovibrio vulgaris*); DD (*Desulfovibrio desulfuricans G20*); GM (*Geobacter metallireducens*); GS (*Geobacter sulfurreducens PCA*); DA (*Desulfuromonas *species); DP (*Desulfotalea psychrophila*).

All δ-proteobacteria studied possess genes for *de novo *biotin synthesis from pimeloyl-CoA precursor (*bioF*, *bioA*, *bioD*, *bioB*) and the bifunctional gene *birA*, but the initial steps of the biotin pathway are variable in these species (Figure 1). The *Geobacter *species have the *bioC-bioH *gene pair, which is required for the synthesis of pimeloyl-CoA in *Escherichia coli*. The *Desulfuromonas *species contain both *bioC-bioH *and *bioW *genes, representing two different pathways of pimeloyl-CoA synthesis. In contrast, *D. psychrophila *is predicted to synthesize a biotin precursor using the *bioC-bioG *gene pair, where the latter gene was only recently predicted to belong to the biotin pathway [20]. Both *Desulfovibrio *species have an extended biotin operon with five new genes related to the fatty-acid biosynthetic pathway. Among these new biotin-regulated genes not present in other δ-proteobacteria studied, there are homologs of acyl carrier protein (ACP), 3-oxoacyl-(ACP) synthase, 3-oxoacyl-(ACP) reductase and hydroxymyristol-(ACP) dehydratase. From positional and regulatory characteristics we conclude that these genes are functionally related to the biotin pathway. The most plausible hypothesis is that they encode a novel pathway for pimeloyl-CoA synthesis, as the known genes for this pathway, *bioC*, *bioH*, *bioG *and *bioW*, are missing in the *Desulfovibrio *species.

#### Riboflavin

Riboflavin (vitamin B_2_) is an essential component of basic metabolism, being a precursor to the coenzymes flavin adenine dinucleotide (FAD) and flavin mononucleotide (FMN). The only known mechanism of regulation of riboflavin biosynthesis is mediated by a conserved RNA structure, the *RFN*-element, which is widely distributed in diverse bacterial species [21]. The δ-proteobacteria in this study possess a conserved gene cluster containing all genes required for the *de novo *synthesis of riboflavin (*ribD-ribE-ribBA-ribH*), but lack this regulatory element. The only exception is *D. psychrophila*, which has an additional gene for 3,4-dihydroxy-2-butanone-4-phosphate synthase (*ribB2*) with an upstream regulatory *RFN *element.

#### Thiamine

**Figure 2:**
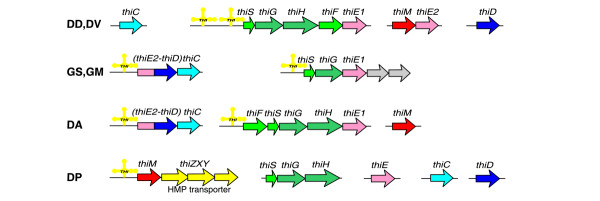
Genomic organization of the thiamin biosynthetic genes and regulatory *THI*-elements (yellow structures). See Figure 1 legend for abbreviations.

Vitamin B_1 _in its active form, thiamine pyrophosphate, is an essential coenzyme synthesized by the coupling of pyrimidine (HMP) and thiazole (HET) moieties in bacteria. The only known mechanism of regulation of thiamine biosynthesis in bacteria is mediated by a conserved RNA structure, the *THI*-element [22]. Search for thiamine-specific regulatory elements in the genomes of δ-proteobacteria identified one or two *THI*-elements per genome that are located upstream of thiamine biosynthetic operons (Figure 1 in Additional data file 1). The δ-proteobacteria possess all the genes required for the *de novo *synthesis of thiamine (Figure 2) with the exception of *Geobacter *species, which lack some genes for the synthesis and salvage of the HET moiety (*thiF*, *thiH *and *thiM*), and *D. psychrophila*, which has no *thiF*. In most δ-proteobacteria there are two paralogs of the thiamine phosphate synthase *thiE*, and *Geobacter *and *Desulfuromonas *species have fused genes *thiED*. In *D. psychrophila*, the only *THI*-regulated operon includes HET kinase *thiM *and previously predicted HMP transporter *thiXYZ *[22], whereas other thiamine biosynthetic genes are not regulated by the *THI*-element (Figure 2).

In most cases, downstream of a *THI*-element there is a candidate terminator hairpin, yielding regulation by the transcription termination/antitermination mechanism. The two exceptions predicted to be involved in translational attenuation are *THI*-elements upstream of genes *thiED *in *Desulfuromonas *and *thiM *in *D. psychrophila*. In the *Desulfovibrio *species, the *thiSGHFE *operon is preceded by two tandem *THI*-elements, each followed by a transcriptional terminator. This is the first example of possible gene regulation by tandem riboswitches.

#### Cobalamin

**Figure 3:**
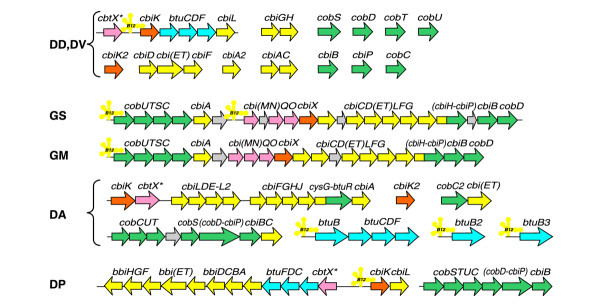
Genomic organization of the cobalamin biosynthetic genes and regulatory *B*12-elements (yellow cloverleaf-type structures). Genes of the first part of the pathway, involved in the corrin ring synthesis are shown as yellow arrows, the genes required for the attachment of the aminopropanol arm and assembly of the nucleotide loop in vitamin B_12 _are in green. Cobalt transporters and chelatases used for the insertion of cobalt ions into the corrin ring are shown in pink and orange, respectively. ABC transport systems for vitamin B_12 _are shown in blue. See Figure 1 legend for abbreviations.

Adenosylcobalamin (Ado-CBL), a derivative of vitamin B_12_, is an essential cofactor for several important enzymes. The studied genomes of δ-proteobacteria possess nearly complete sets of genes required for the *de novo *synthesis of Ado-CBL (Figure 3). The only exception is the precorrin-6x reductase, *cbiJ*, which was found only in *Desulfuromonas *but not in other species. The occurrence of CbiD/CbiG enzymes instead of the oxygen-dependent CobG/CobF ones suggests that these bacteria, consistent with their anaerobic lifestyle, use the anaerobic pathway for B_12 _synthesis similar to that used by *Salmonella typhimurium *[23].

Ado-CBL is known to repress expression of genes for vitamin B_12 _biosynthesis and transport via a co- or post-transcriptional regulatory mechanism, which involves direct binding of Ado-CBL to the riboswitch called the *B12*-element [24,25]. A search for *B12*-elements in the genomes of δ-proteobacteria produced one *B12*-element in *D. desulfuricans*, *D. psychrophila *and *G. metallireducens*, two in *D. vulgaris *and *G. sulfurreducens*, and four in *Desulfuromonas *(Figure 2 in Additional data file 1). In *Geobacter *species these riboswitches regulate a large locus containing almost all the genes for the synthesis of Ado-CBL (Figure 3). One *B12*-element in the *Desulfovibrio *species regulates both the cobalamin-synthesis genes *cbiK-cbiL *and the vitamin B_12 _transport system *btuCDF*, whereas three such regulatory elements in *Desulfuromonas *precede different vitamin B_12 _transport loci. In *D. psychrophila*, a *B12*-element occurs within a large B_12 _synthesis gene cluster and precedes the *cbiK-cbiL *genes.

The most interesting observation is that genes encoding the B_12_-independent ribonucleotide reductase NrdDG are preceded by *B12*-elements in *D. vulgaris *and *Desulfuromonas*. Notably, all δ-proteobacteria have another type of ribonucleotide reductase, NrdJ, which is a vitamin B_12_-dependent enzyme. We propose that when vitamin B_12 _is present in the cell, expression of the B_12_-independent isozyme is inhibited, and a relatively more efficient B_12_-dependent isozyme is used. This phenomenon has been previously observed in other bacterial genomes [26].

#### Methionine

**Figure 4:**
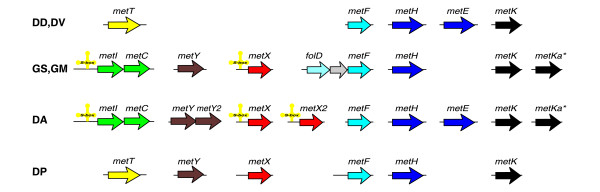
Genomic organization of the methionine biosynthetic genes and regulatory S-boxes (yellow cloverleaf-type structures). See Figure 1 legend for abbreviations.

The sulfur-containing amino acid methionine and its derivative *S*-adenosylmethionine (SAM) are important in protein synthesis and cellular metabolism. There are two alternative pathways for methionine synthesis in microorganisms, which differ in the source of sulfur. The *trans*-sulfuration pathway (*metI-metC*) utilizes cysteine, whereas the direct sulfhydrylation pathway (*metY*) uses inorganic sulfur instead. All δ-proteobacteria in this study except the *Desulfovibrio *species possess a complete set of genes required for the *de novo *synthesis of methionine (Figure 4). The *Geobacter *species and possibly *Desulfuromonas *have some redundancy in the pathway. First, these genomes contain the genes for both alternative pathways of the methionine synthesis. Second, they possess two different SAM synthase isozymes, classical bacterial-type MetK and an additional archaeal-type enzyme [27]. Moreover, it should be noted that the B_12_-dependent methionine synthase MetH in these bacteria lacks the carboxy-terminal domain, which is involved in reactivation of spontaneously oxidized coenzyme B_12_.

In Gram-positive bacteria, SAM is known to repress expression of genes for methionine biosynthesis and transport via direct binding to the S-box riboswitch [28]. In contrast, Gram-negative enterobacteria control methionine metabolism using the SAM-responsive transcriptional repressor MetJ. The δ-proteobacteria in this study have no orthologs of MetJ, but instead, we identified S-box regulatory elements upstream of the *metIC *and *metX *genes in the genomes of the *Geobacter *species and *Desulfuromonas *(see Figure 3 in Additional data file 1). A strong hairpin with a poly(T) region follows all these S-boxes, implying involvement of these S-boxes in a transcriptional termination/antitermination mechanism.

Both *Desulfovibrio *species have genes involved in the conversion of homocysteine into methionine (*metE*, *metH *and *metF*), which could be involved in the SAM recycling pathway, but not those genes required for *de novo *methionine biosynthesis. The ABC-type methionine transport system (*metNIQ*), which is widely distributed among bacteria, was also not found in these δ-proteobacteria. The *Desulfovibrio *species appear to have the single-component methionine transporter *metT *[28].

#### Lysine

The amino acid lysine is produced from aspartate through the diaminopimelate (DAP) pathway in most bacteria. The first two stages of the DAP pathway, catalyzed by aspartokinase and aspartate semialdehyde dehydrogenase, are common for the biosynthesis of lysine, threonine, and methionine. The corresponding genes were found in δ-proteobacteria where they form parts of different metabolic operons. Four genes for the conserved stages of the lysine synthesis pathway (*dapA*, *dapB*, *dapF *and *lysA*) were further identified in δ-proteobacteria, whereas we did not find orthologs for three other genes (*dapC*, *dapE *and *dapD*), which vary in bacteria using different meso-DAP synthesis pathways. The lysine synthesis genes are mostly scattered along the chromosome, and in only some cases are *dapA *and either *dapB*, *dapF *or *lysA *clustered. All δ-proteobacteria studied lack the previously known lysine transporter LysP. However, in *D. desulfuricans *and *D. psychrophila *we found a gene for another candidate lysine transporter, named *lysW*, which was predicted in our previous genomic survey [29].

In various bacterial species, lysine is known to repress expression of genes for lysine biosynthesis and transport via the L-box riboswitch [30]. In addition, Gram-negative enterobacteria use the lysine-responsive transcriptional factor LysR for control of the *lysA *gene. Among the δ-proteobacteria studied, we found neither orthologs of LysR, nor representatives of the L-box RNA regulatory element. In an attempt to analyze potential lysine regulons in this phylogenetic group, we collected upstream regions of all lysine biosythesis genes and applied SignalX as a signal detection procedure [31]. The strongest signal, a 20-bp palindrome with consensus GTGGTACTNNNNAGTACCAC, was observed upstream of the *lysX-lysA *operons in both *Desulfovibrio *genomes and the candidate lysine transporter gene *lysW *in *D. desulfuricans *(Table 2). The first gene in this operon, named *lysX*, encodes a hypothetical transcriptional regulator with a helix-turn-helix motif (COG1378) and is the most likely candidate for the lysine-specific regulator role in *Desulfovibrio*. To find new members of the regulon, the derived profile (named LYS-box) was used to scan the *Desulfovibrio *genomes. The lysine regulon in these genomes appears to include an additional gene (206613 in *D. vulgaris*, and 394397 in *D. desulfuricans*), which encodes an uncharacterized membrane protein with 14 predicted transmembrane segments. We predict that this new member of the lysine regulon might be involved in the uptake of lysine or some lysine precursor.

### Metal ion homeostasis

#### Iron

Iron is necessary for the growth of most bacteria as it participates in many major biological processes [32]. In aerobic environments, iron is mainly insoluble, and microorganisms acquire it by secretion and active transport of high-affinity Fe(III) chelators. Under anaerobic conditions, Fe(II) predominates over ferric iron, and can be transported by the ATP-dependent ferrous iron transport system FeoAB. Genomes of anaerobic δ-proteobacteria contain multiple copies of the *feoAB *genes, and lack ABC transporters for siderophores. Regulation of iron metabolism in bacteria is mediated by the ferric-uptake regulator protein (FUR), which represses transcription upon interaction with ferrous ions. FUR can be divided into two domains, an amino-terminal DNA-binding domain and a carboxy-terminal Fe(II)-binding domain. The consensus binding site of *E. coli *FUR is a palindromic sequence GATAATGATNATCATTATC [33].

**Figure 5:**
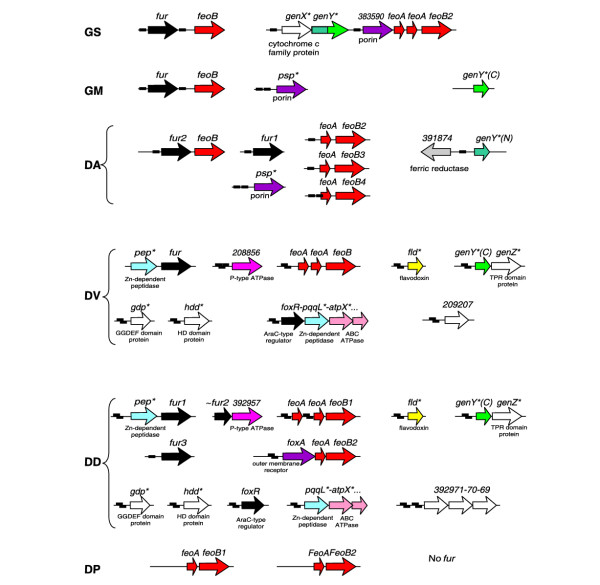
Genomic organization of the predicted iron-regulated genes and FUR-binding sites (small black rectangles). *Name introduced in this study. See Figure 1 legend for abbreviations.

In all δ-proteobacteria studied except *D. psychrophila*, we identified one to three FUR orthologs that form a distinct branch (FUR_Delta) in the phylogenetic tree of the FUR/ZUR/PerR protein family (see below). One protein, FUR2 in *D. desulfuricans*, lacks an amino-terminal DNA-binding domain and is either non-functional or is involved in indirect regulation by forming inactive heterodimers with two other FUR proteins. Scanning the genomes with the FUR-box profile of *E. coli *did not result in identification of candidate FUR-boxes in δ-proteobacteria. In an attempt to analyze potential iron regulons in this phylogenetic group, we collected upstream regions of the iron-transporter genes *feoAB *and applied SignalX to detect regulatory signals. The strongest signal, a 17-bp palindrome with consensus WTGAAAATNATTTTCAW (where W indicates A or T), was observed upstream of the multiple *feoAB *operons and *fur *genes in all δ-proteobacteria except *D. psychrophila *(Table 3). The constructed search profile (dFUR-box) was applied to detect new candidate FUR-binding sites in these five genomes (Figure 5 and Table 3).

The smallest FUR regulons were observed in the *Geobacter *and *Desulfuromonas *species, where they include the ferrous iron transporters *feoAB *(one to four copies per genome), the *fur *genes themselves (one copy in the *Geobacter *species and two copies in *Desulfuromonas*), and two hypothetical porins. The first one, named *psp*, was found only in *G. metallireducens *and *Desulfuromonas *genomes, where it is preceded by two tandem FUR-boxes. The *psp *gene has homologs only in *Aquifex aeolicus *and in various uncultured bacteria, and in one of them (a β-proteobacterium) it is also preceded by two FUR-boxes (GenBank entry AAR38161.1). This gene is weakly similar to the family of phosphate-selective porins (PFAM: PF07396) from various Gram-negative bacteria. The second hypothetical porin was found only in *G. sulfurreducens *(383590), where it is preceded by a FUR-box and followed by *feoAB *transporter. This gene, absent in other δ-proteobacteria, has only weak homologs in some Gram-negative bacteria and belongs to the carbohydrate-selective porin OprB family (PFAM: PF04966). Thus, two novel genes predicted to fall under FUR control encode hypothetical porins that could be involved in ferrous iron transport.

Another strong FUR-box in the *G. sulfurreducens *genome precedes a cluster of two hypothetical genes located immediately upstream of the *feoAB*-containing operon. The first gene in this operon, named *genX *(383594), has no orthologs in other bacteria and the encoded protein has a heme-binding site signature of the cytochrome *c *family (PFAM: PF00034). The second gene, named *genY *(383592), encodes a two-domain protein that is not similar to any known protein. In *Desulfuromonas*, an ortholog of the *genY *amino-terminal domain (391875) is divergently transcribed from a predicted ferric reductase (391874), and their common upstream region contains a strong FUR-box. Moreover, orthologs of the *genY *C-terminal domain were identified in *Desulfovibrio *species, where they are again preceded by two tandem FUR-boxes and form a cluster with the hypothetical gene, *genZ*, encoding a protein of 100 amino acids with two tetratricopeptide repeat domains that are usually involved in protein-protein interactions (PFAM: PF00515). From genomic analysis alone it is difficult to predict possible functions of these new members of the FUR regulon in δ-proteobacteria.

Two *Desulfovibrio *species have significantly extended FUR regulons that are largely conserved in these genomes and include ferrous iron transporter genes *feoAB *and many hypothetical genes. Another distinctive feature of the FUR regulon in *Desulfovibrio *species is a structure of two partially overlapping FUR-boxes shifted by 6 bp. Interestingly, the flavodoxin gene, *fld*, is predicted to be regulated by FUR in both *Desulfovibrio *species. In addition to this iron-repressed flavodoxin (a flavin-containing electron carrier), the *Desulfovibrio *species have numerous ferredoxins (an iron-sulfur-containing electron carrier). One possible explanation is that in iron-restricted conditions these microorganisms can replace ferredoxins with less-efficient, but iron-independent alternatives. A similar regulatory strategy has been previously described for superoxide dismutases in *E. coli*, *Bordetella pertusis *and *Pseudomonas aeruginosa *[34-36] and predicted, in a different metabolic context, for B_12_-dependent and B_12_-independent enzymes [26]; see the discussion above.

Other predicted regulon members with conserved FUR-boxes in both *Desulfovibrio *species are the hypothetical genes *pep *(Zn-dependent peptidase), *gdp *(GGDEF domain protein, PF00990), *hdd *(metal dependent HD-domain protein, PF01966), and a hypothetical P-type ATPase (392971) that could be involved in cation transport, and a long gene cluster starting from the *pqqL *gene (Zn-dependent peptidase). The latter cluster contains at least 10 hypothetical genes encoding components of ABC transporters and biopolymer transport proteins (*exbB*, *exbD *and *tonB*). In *D. vulgaris*, the first gene in this FUR-regulated cluster is an AraC-type regulator named *foxR*, since it is homologous to numerous FUR-controlled regulators from other genomes (*foxR *from *Salmonella typhi*, *alcR *from *Bordetella pertussis*, *ybtA *from *Yersinia *species, *pchR *from *Pseudomonas aeruginosa*), which usually regulate iron-siderophore biosynthesis/transport operons [33]. An ortholog of *foxR*, a single FUR-regulated gene, was identified in *D. desulfuricans *located about 30 kb away from the FUR-regulated *pqqL *gene cluster. Given these observations, we propose that this gene cluster is involved in siderophore transport and is regulated by FoxR.

A hypothetical gene in *D. vulgaris *(*209207*) has the strongest FUR-box in this genome; however, its orthologs in *D. desulfuricans *are not predicted to belong to the FUR regulon. Another operon in *D. desulfuricans *(392971-392970-392969), encoding three hypothetical proteins, is preceded by two candidate FUR-boxes, but these genes have no orthologs in other δ-proteobacteria. Thus, FUR-dependent regulation of these hypothetical genes is not confirmed in other species, and their possible role in the iron homeostasis is not clear.

#### Nickel

The transition metal nickel (Ni) is an essential cofactor for a number of prokaryotic enzymes, such as [NiFe]-hydrogenase, urease, and carbon monoxide dehydrogenase (CODH). Two major types of nickel-specific bacterial transporters are represented by the NikABCD system of *E. coli *(the nickel/peptide ABC transporter family) and the HoxN of *Ralstonia eutropha *(the NiCoT family of nickel/cobalt permeases). Nickel uptake must be tightly regulated because excessive nickel is toxic. In *E. coli *and some other proteobacteria, nickel concentrations are controlled by transcriptional repression of the *nikABCD *operon by the Ni-dependent regulator NikR [37].

**Figure 6:**
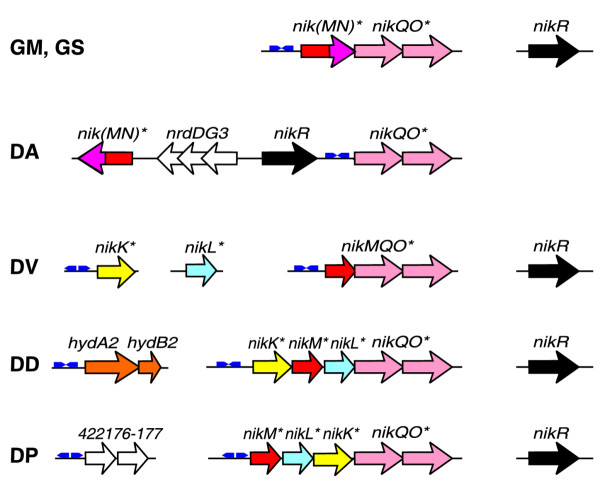
Genomic organization of the nickel-regulated genes and NikR-binding sites (small blue arrows). See Figure 1 legend for abbreviations.

The genomes of δ-proteobacteria studied so far contain multiple operons encoding [NiFe] and [Fe] hydrogenases and Ni-dependent CODH, but lack urease genes. Both known types of nickel-specific transporters are absent in δ-proteobacteria, but these genomes contain orthologs of the nickel repressor *nikR*. In an attempt to identify potential nickel transporters in this taxonomic group, we analyzed the genome context of the *nikR *genes. The *nikR *gene in *Desulfuromonas *is co-localized with a hypothetical ABC transport system, which is weakly homologous to the cobalt ABC-transporter *cbiMNQO *from various bacteria. Orthologs of this system, named here *nikMNQO*, are often localized in proximity to Ni-dependent hydrogenase or urease gene clusters in various proteobacteria (data not shown). Among δ-proteobacteria, the *Geobacter *species have a complete *nikMNQO *operon, whereas operons in *D. desulfuricans *and *D. psychrophila *lack the *nikN *component but include two additional genes, named *nikK *and *nikL*, which both encode hypothetical proteins with amino-terminal transmembrane segments (Figure 6). *Desulfovibrio vulgaris *has a *nikMQO *cluster and separately located *nikK *and *nikL *genes. Since various other proteobacteria also have the same clusters including *nikK *and *nikL*, but not *nikN *(data not shown), we propose that these two genes encode additional periplasmic components of the NikMQO ABC transporter, possibly involved in the nickel binding.

By applying SignalX to a set of upstream regions of the *nikMQO *operons, we identified *de novo *the NikR binding signal in all δ-proteobacteria except *D. psychrophila *(Table 4). This signal has the same structure as in enterobacteria (an inverted repeat of 27-28 bp), but its consensus (GTGTTAC-[N_13/14_]-GTAACAC) differs significantly from the consensus of NikR binding signal of enterobacteria (GTATGAT-[N_13/14_]-ATCATAC) [37]. Using the derived profile to scan the genomes of δ-proteobacteria we identified one more candidate NikR-binding site in *D. desulfuricans*. Thus the nickel regulon in this bacterium includes the *hydAB2 *operon, encoding periplasmic iron-only hydrogenase. Altogether, *D. desulfuricas *has three paralogs of [NiFe] hydrogenase and two paralogs of [Fe] hydrogenase. We predict that an excess of nickel represses a nickel-independent hydrogenase isozyme using the Ni-responsive repressor NikR. Regulation of hydrogenase enzymes by NikR has not been described previously. A closer look at the upstream region of the putative nickel transport operon in *D. psychrophila *revealed similar NikR consensus half-sites but in the opposite orientation to each other (GTAACAC-[N_13/14_]-GTGTTAC). Searching the genomes with this reversed NikR signal, we observed one more hypothetical gene cluster in *D. psychrophila *which has two high-scoring NikR-sites in the upstream region, and a NikR-site upstream of the single *nikK *gene in *D. vulgaris *(Figure 6).

#### Zinc

**Figure 7:**
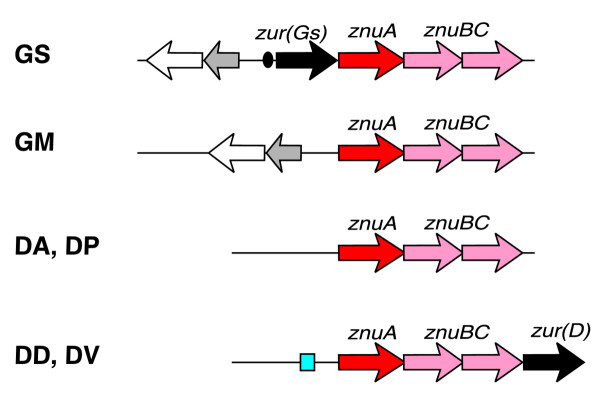
Genomic organization of predicted zinc ABC transporters and ZUR-binding sites. The black oval and blue box represent two different types of ZUR-binding site. See Figure 1 legend for abbreviations.

Zinc is an important component of many proteins, but in large concentrations it is toxic to the cell. Thus zinc repressors ZUR regulate high-affinity zinc transporters *znuABC *in various bacteria [38]. An orthologous zinc transporter was found in δ-proteobacteria (Figure 7). In *G. sulfurreducens *and the *Desulfovibrio *species, this cluster also includes a hypothetical regulatory gene from the FUR/ZUR/PerR family, named *zur_Gs *and *zur_D*, respectively. Phylogenetic analysis of this protein family demonstrated that ZUR_Gs and ZUR_D are not close relatives and are only weakly similar to known FUR, ZUR, and PerR regulators from other bacteria (see below). The predicted ZUR-binding site located just upstream of the *zur-znuABC *operon in *G. sulfurreducens *is highly similar to the ZUR consensus of Gram-positive bacteria (TAAATCGTAATNATTACGATTTA). Another strong signal, a 17-bp palindrome with consensus ATGCAACNNNGTTGCAT, was identified upstream of the *znuABC-zur *operons in two *Desulfovibrio *genomes (Table 5). Although *znuABC *genes are present in all δ-proteobacteria, we observed neither candidate ZUR regulators, nor ZUR-binding sites in *G. metallireducens*, *Desulfuromonas *and *D. psychrophila*, suggesting either the absence of zinc-specific regulation or presence of another regulatory mechanism for these genes.

#### Cobalt

The previously described cobalt transport system CbiMNQO was found only in the *Geobacter *species, where it is located within the B_12_-regulated *cbi *gene cluster close to the cobaltochelatase gene *cbiX*, responsible for incorporation of cobalt ions into the corrin ring (see the 'Cobalamin' section above). In contrast, other δ-proteobacteria, possessing a different cobaltochelatase (*cbiK*), lack homologs of any known cobalt transporter. It was previously suggested by global analysis of the B_12 _metabolism that different types of cobalt transporters are interchangeable in various bacterial species [26]. From genome context analysis and positional clustering with the *cbiK *gene, we predicted a novel candidate cobalt transporter in δ-proteobacteria, named *cbtX *(Figure 3), which was previously annotated as a hypothetical transmembrane protein conserved only in some species of archaea (COG3366).

#### Molybdenum

**Figure 8:**
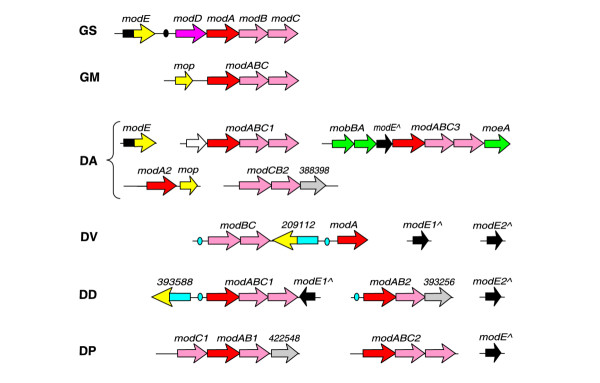
Genomic organization of predicted molybdate ABC transporters and ModE-binding sites (small ovals). The black and blue ovals represent two different types of ModE-binding site. See Figure 1 legend for abbreviations.

Molybdenum (Mo) is another transition metal essential for bacterial metabolism. Bacteria take up molybdate ions via a specific ABC transport system encoded by *modABC *genes. Mo homeostasis is regulated by the molybdate-responsive transcription factor ModE, containing an amino-terminal DNA-binding domain and two tandem molybdate-binding domains. Orthologs of ModE are widespread among prokaryotes, but not ubiquitous [39]. All δ-proteobacteria have one or more homologs of the *modABC *transporter (Figure 8). However, full-length *modE *genes containing both DNA- and molybdate-binding domains were observed only in *G. sulfurreducens *and *Desulfuromonas*. In *G. sulfurreducens*, the molybdate transport operon is co-localized with *modE *and is preceded by a putative ModE-binding site (Table 6), which is similar to the *E. coli *consensus of ModE (ATCGNTATATA-[N_6_]-TATATANCGAT). In contrast, we could not identify *E. coli*-type ModE-binding sites upstream of the *mod *operons in *Desulfuromonas*, indicating that these operons may be regulated by a different, unidentified signal.

Three other δ-proteobacteria (two *Desulfovibrio *species and *D. psychrophila*) have genes encoding a single DNA-binding domain of ModE (Figure 8). Searching with the *E. coli*-type profile did not reveal candidate binding sites of ModE in these species. To predict potential ModE sites *de novo*, we collected upstream regions of all molybdate transport operons and applied SignalX. In both *Desulfovibrio *genomes, we identified a common inverted repeat with consensus CGGTCACG-[N_14_]-CGTGACCG, which is considerably different from the *E. coli *consensus of ModE (Table 6 and Figure 8). The *modABC *gene cluster in these species includes an additional chimeric gene encoding a fusion of phage integrase family domain (PF00589) and one or two molybdate-binding domains (MOP). The functions of these chimeric molybdate-binding proteins, and the mechanism of Mo-sensing by DNA-binding ModE domains in the *Desulfovibrio *species, are not clear.

### Stress response regulons

#### Oxidative stress

Under aerobic conditions, generation of highly toxic and reactive oxygen species such as superoxide anion, hydrogen peroxide and the hydroxyl radical leads to oxidative stress with deleterious effects [40]. Strictly anaerobic sulfate-reducing bacteria are adapted to survive in transient oxygen-containing environments by intracellular reduction of oxygen to water using rubredoxin:oxygen oxidoreductase (Roo) as the terminal oxidase [41]. The main detoxification system for reactive oxygen species in aerobic and anaerobic bacteria involves superoxide dismutase (Sod), catalase (KatA, KatG) and nonspecific peroxidases (for example, AhpC). In addition to these enzymes, *Desulfovibrio *species have an alternative mechanism for protecting against oxidative stress, which includes rubredoxin oxidoreductase (Rbo), which has superoxide reductase activity, rubrerythrin (Rbr) with NADH peroxidase activity, and rubredoxin-like proteins (Rub, Rdl), which are used as common intermediary electron donors [42].

**Figure 9:**
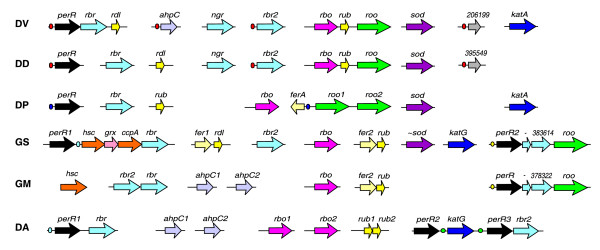
Genomic organization of genes involved in oxidative stress response. Dots of various colors represent predicted PerR-binding sites with different consensus sequences. See Figure 1 legend for abbreviations.

**Figure 10:**
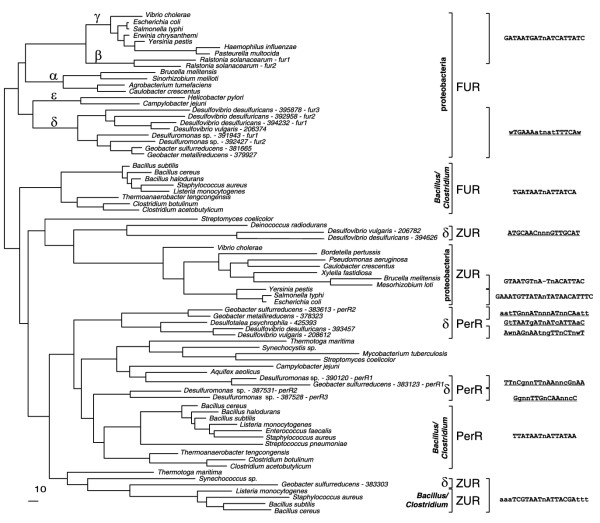
Maximum-likelihood phylogenetic tree of the FUR/ZUR/PerR family of transcriptional regulators. Consensus sequences of binding sites predicted in this study are underlined. See Figure 1 legend for abbreviations.

Searching for orthologs of the oxidative stress-related genes in the genomes in this study revealed great variability in content and genomic organization (Figure 9). We also searched for homologs of transcription factors known to be involved in regulation of the peroxide and superoxide stress responses. Lacking orthologs of the *E. coli *OxyR and SoxR/SoxS regulators, the δ-proteobacteria studied have instead multiple homologs of the peroxide-sensing regulator PerR of *B. subtilis *[43]. The PerR-specific branch on the phylogenetic tree of the FUR/ZUR/PerR family contains at least three distinct sub-branches with representatives from δ-proteobacteria (Figure 10). In all cases except *D. psychrophila*, the *perR *genes are co-localized on the chromosome with various peroxide stress-responsive genes (Figure 9). However, the upstream regions of these genes contain no candidate PerR-binding sites conforming to the *B. subtilis *PerR consensus TTATAATNATTATAA. Applying the SignalX program to various subsets of upstream regions of peroxide stress-responsive genes resulted in identification of candidate PerR operators in δ-proteobacteria (Table 7).

In the *Desulfovibrio *species, a common palindromic signal was found upstream of the *perR *and *rbr2 *genes. In *D. vulgaris*, *perR *forms an operon with *rbr *and *rdl *genes [42]. Searching for genes with the derived profile identified additional candidate members of the PerR regulon, alkyl hydroperoxide reductase *ahpC *in *D. vulgaris *(*D. desulfuricans *has no ortholog of *ahpC*), and a hypothetical gene of unknown function in both *Desulfovibrio *species (206199 in *D. vulgaris *and 395549 in *D. desulfuricans*).

**Figure 11:**
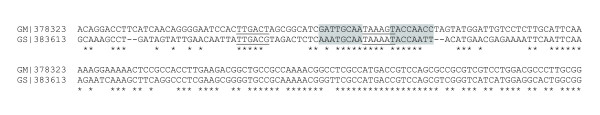
Pairwise sequence alignment of upstream regions of the *perR-rbr-roo *operons from *Geobacter *species. Conserved palindromic signal, that is the candidate PerR-box, is highlighted in gray. Predicted SD-boxes and start codons of the *perR *genes are in bold. Predicted -10 and -35 promoter boxes are underlined. *Conserved position of alignment. See Figure 1 legend for abbreviations.

The *perR-rbr-roo *operon in both *Geobacter *species is preceded by a conserved palindromic region (Table 7) which overlaps a candidate -10 promoter element (Figure 11). The second *perR *paralog in *G. sulfurreducens *(named *perR2*), which is followed by a gene cluster containing two cytochrome peroxidase homologs (*hsc *and *ccpA*), glutaredoxin (*grx*) and rubrerythrin (*rbr*), has a close ortholog in the *Desulfuromonas *species, where it precedes the *rbr *gene (Figures 9, 10). For these gene clusters we found a common palindromic signal, which is not similar to other predicted PerR signals in δ-proteobacteria (Table 7). Two other *perR *paralogs in *Desulfuromonas *(*perR2 *and *perR3*) probably result from a recent gene duplication (Figure 10), and both are co-localized on the chromosome with the peroxide stress-responsive genes *katG *and *rbr2*, respectively (Figure 9). A common new signal identified upstream of the *katG *and *perR3 *genes is probably recognized by both PerR2 and PerR3 regulators in this organism (Table 7).

The PerR regulons in δ-proteobacteria are predicted to include only a small subset of all peroxide stress-related genes identified in these genomes. In addition to the mainly local character of the predicted regulation, these regulons seem to be highly variable between different species, both in their content and DNA signals.

#### Heat shock

In bacteria, two major mechanisms regulating expression of heat-shock proteins are positive control by alternative sigma factor σ^32^, encoded by the *rpoH *gene, and negative control by binding of the repressor protein HrcA to palindromic operators with a consensus TTAGCACTC-[N_9_]-GAGTGCTAA called CIRCE [44]. The *rpoH *gene was identified in the genomes of all δ-proteobacteria studied. Though the HrcA/CIRCE system is conserved in very diverse taxonomic groups of bacteria, it is not universal, as some γ-proteobacteria lack it [45]. We detected the *hrcA *genes and CIRCE sites in all genomes studied except *D. psychrophila *(Table 8).

We then searched the genomes of δ-proteobacteria with previously constructed profiles for σ^32 ^promoters and CIRCE [45]. As was observed previously for other bacteria, the only constant member of the HrcA regulon in δ-proteobacteria is the *groESL *operon. In addition, CIRCE sites are present upstream of the *hrcA-grpE-dnaKJ *operons in the *Geobacter *and *Desulfuromonas *species and upstream of the *rpoH *gene in *G. sulfurreducens*. In contrast to the highly conserved CIRCE signal, the σ^32 ^promoters identified in multiple copies in various proteobacteria are less conserved [45,46]. Among δ-proteobacteria, we identified σ^32^-like promoters upstream of some heat-shock-related genes encoding chaperons (GroE, DnaJ, DnaK, GrpE) and proteases (ClpA, ClpP, ClpX, Lon) (Table 9). Thus, in δ-proteobacteria, as in most proteobacteria, σ^32 ^plays a central part in the regulation of the heat-shock response, although detailed regulatory strategies seem to vary in different species. The alternative HrcA/CIRCE system controls expression of *groE *and other major chaperons.

### Central energy metabolism

#### The CooA regulon for carbon monoxide utilization in *Desulfovibrio *species

**Figure 12:**
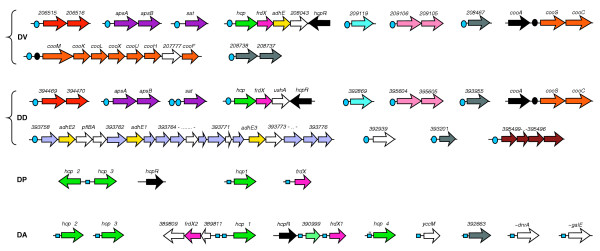
Genomic organization of genes predicted to be regulated by two transcription factors from the CRP/FNR-family. Black circles denote operators for the CO-responsive regulator CooA. Blue circles and squares denote predicted sites of the hypothetical transcriptional factor HcpR with two different consensus sequences, respectively. w, HcpR site with a weak score; ..., a set of gene names that are not shown. See Figure 1 legend for abbreviations.

Growth using carbon monoxide (CO) as the sole energy source involves two key enzymes in the γ-proteobacterium *Rhodospirillum rubrum *- CO dehydrogenase (CODH) and an associated hydrogenase - which are encoded in the *coo *operons and induced by the CO-sensing transcriptional activator CooA [47]. Among the sequenced δ-proteobacteria, only *Desulfovibrio *species have *coo *operons and the CooA regulator. *D. vulgaris *has two separate operons encoding CODH and the associated hydrogenase, whereas *D. desulfuricans *has only one operon encoding CODH (Figure 12). The strongest identified signal, a 16-bp palindrome with consensus TGTCGGCNNGCCGACA, was identified upstream of the *coo *operons from both *Desulfovibrio *species and *R. rubrum *(Table 10a). This consensus conforms to the experimentally known CooA-binding region at the *R. rubrum cooFSCTJ *operon [48].

#### New CRP/FNR-like regulon for sulfate reduction and prismane genes

Sulfate-reducing bacteria are characterized by their ability to utilize sulfate as a terminal electron acceptor. To try to identify the regulatory signals responsible for this metabolism, we applied the signal detection procedure SignalX to a set of upstream regions of genes involved in the sulfate-reduction pathway in *Desulfovibrio *species. A conserved palindromic signal with consensus sequence TTGTGANNNNNNTCACAA was detected upstream of the *sat *and *apsAB *operons, which encode ATP sulfurylase and APS reductase, respectively. This novel signal is identical to the *E. coli *CRP consensus, and we hypothesized that a CRP-like regulator might control the sulfate-reduction regulon in *Desulfovibrio*. Scanning the *Desulfovibrio *genomes resulted in identification of similar sites upstream of many hypothetical genes encoding various enzymes and regulatory systems (Table 10b and Figure 12). One of them, the *hcp *gene in *D. vulgaris*, encodes a hybrid-cluster protein (previously called the prismane-containing protein) of unknown function [49], which is coexpressed with a hypothetical ferredoxin gene, named frdX*: new gene names introduced in this study are marked by asterisk. In both *Desulfovibrio *species, the hcp-frdX* genes are co-localized with a hypothetical regulatory gene from the CRP/FNR family of transcriptional regulators, named HcpR* for the Hcp regulator (Figure 12).

Close HcpR* orthologs were detected in two other δ-proteobacteria, *D. psychrophila *and *Desulfuromonas*; however, the same CRP-like signals were not present in their genomes. Examination of a multiple alignment of the CRP/FNR-like proteins revealed one specific amino acid (Arg 180) in the helix-turn-helix motif involved in DNA recognition, which is changed from arginine (for example, in *E. coli *CRP and *Desulfovibrio *HcpR*) to serine and proline in these two δ-proteobacteria (data not shown). As both these species have multiple *hcp *and *frdX *paralogs, we applied SignalX to a set of corresponding upstream regions and obtained another FNR-like palindromic signal with consensus at ATTTGACCNNGGTCAAAT, which is notably distinct from the CRP-like signal in the third position (which has T instead of G). Such candidate sites were observed upstream of all *hcp *and *frdX *paralogs identified in *D. psychrophila *and *Desulfuromonas*, as well as upstream of some additional genes in *Desulfuromonas*, for example those encoding polyferredoxin and cytochrome *c *heme-binding protein (Table 10 and Figure 12).

The HcpR regulon was also identified in other taxonomic groups, including *Clostridium*, *Thermotoga*, *Bacteroides*, *Treponema *and *Acidothiobacillus *species, and in all cases candidate HcpR sites precede *hcp *orthologs (data not shown). Moreover, the *hcpR *gene is often co-localized with *hcp *on the chromosome. In clostridia, *frdX *orthologs are also preceded by candidate HcpR sites. These data indicate that the main role of HcpR is control of expression of two hypothetical proteins - hybrid-cluster protein and ferredoxin - which are most probably involved in electron transport. However, the HcpR regulon is significantly extended in some organisms. Additional members of this regulon that are conserved between the two *Desulfovibrio *species include two operons involved in sulfate reduction (*apsAB *and *sat*), a hypothetical cluster of genes (206515-206516) with similarity to dissimilative sulfite and nitrite reductases, polyferredoxin, a hypothetical gene conserved in Archaea (209119), and the putative thiosulfate reductase operon *phcAB *(209106-209105). Notably, both CooA and HcpR candidate sites precede the *cooMKLXUHF *operon for CODH-associated hydrogenase, which is present only in *D. vulgaris*.

**Figure 13:**
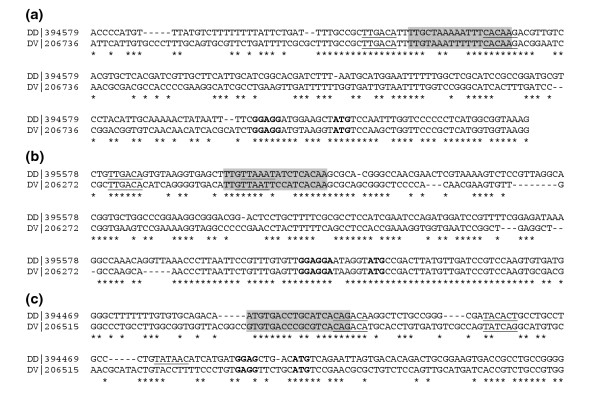
Pairwise sequence alignment of upstream regions of the predicted HcpR-regulated operons from *Desulfovibrio *species. **(a) ***sat*; **(b) ***apsAB*; **(c) ***206515-206516*. Candidate HcpR sites are highlighted in gray. Predicted SD-boxes and start codons of the first genes in the operons are in bold. Predicted '-10' and '-35' promoter boxes are underlined. *Conserved position of alignment. See Figure 1 legend for abbreviations.

Because regulators from the CRP/FNR family are able to both repress and activate gene expression, it was interesting to predict the mode of regulation of the HcpR regulon members. To this end, we investigated the positions of candidate HcpR sites in pairwise alignments of orthologous regulatory regions from the two *Desulfovibrio *species. These two closely related genomes are diverse enough to identify regulatory elements as conserved islands in alignments of intergenic regions. For the *sat *and *apsAB *operons, the HcpR sites were found within highly conserved parts of alignments and in both cases the site overlaps the -10 box of a site strongly resembling a promoter (Figure 13a,b), suggesting repression of the genes by HcpR. In contrast, positive regulation by HcpR could be proposed for the *hcp-frdX*, 206515-206516 and 209119 operons, which have HcpR sites upstream or slightly overlapping the -35 box of predicted promoters (Figure 13c). In the case of the *cooMKLXUHF *operon in *D. vulgaris*, the HcpR site is located upstream of the candidate site of the known positive regulator CooA; thus it is also predicted to be an activator site.

By analysis of the functions of genes co-regulated by HcpR, it is difficult to predict the effector for this novel regulon. The physiological role of the hybrid iron-sulfur cluster protein Hcp, the most conserved member of the HcpR regulon, is not yet characterized despite its known three-dimensional structure and expression profiling in various organisms. In two facultative anaerobic bacteria, *E. coli *and *Shewanella oneidensis*, the *hcp *gene is expressed only under anaerobic conditions in the presence of either nitrate or nitrite as terminal electron acceptors [50,51]. More recent expression data obtained for anaerobic *D. vulgaris *have showed strong upregulation of the hcp-frdX* and 206515-206516 operons by nitrite stress (J. Zhou, personal communication). While HcpR is predicted to activate these two hypothetical operons, as well as the CODH-associated hydrogenase operon, it most probably represses two enzymes from the sulfate reduction pathway, APS reductase and ATP sulfurylase. We hypothesize that HcpR is a key regulator of the energy metabolism in anaerobic bacteria, possibly controlling the transition between utilization of alternative electron acceptors, such as sulfate and nitrate. The absence of the dissimilatory sulfite reductase DsrAB in the predicted HcpR regulon of *Desulfovibrio *could be explained by its experimentally defined ability to reduce both sulfite and nitrite [52].

## Discussion

### Regulation of biosynthesis pathways

Because the organisms considered in this study are commonly identified on the basis of their catabolic capabilities, comparatively little is known about the regulation of their biosynthetic pathways. In this study, we identified a number of previously characterized regulatory mechanisms (involved in biotin, thiamine, cobalamin and methionine synthesis), all of which, excluding the biotin regulon, are mediated by direct interaction of a metabolic product with a riboswitch control element (summarized in Table 11). Of particular interest in this set was observation of a dual tandem *THI*-element riboswitch in *Desulfovibrio *species. Multiple protein-binding sites are a common regulatory feature and often imply cooperative binding of multiple protein factors. Although true riboswitch units do not interact with *trans*-acting factors, it is theoretically possible for independently acting sites to yield a cooperative effect when ligand binding derepresses transcription. For switches that are repressed by ligand binding, however, tandem sites would simply lower the concentration threshold at which a response is seen, but not affect cooperativity unless some more complicated interaction of the sites were allowed. On the one hand, independently acting sites is a simpler mechanism to explain, while on the other hand, it seems unusual that duplicate sites would have evolved to adjust the concentration response instead of simply changing the binding affinity for the ligand at the sequence level. Moreover, it seems unlikely that a tandem switch would be preserved across a large evolutionary distance without offering some other advantage such as cooperativity. It would be interesting to investigate the biochemical behavior of these tandem *THI*-elements in the laboratory to resolve whether their genomic organization reflects a more sophisticated mode of regulation, or is simply an evolutionarily convenient way to adjust the concentration response, or is perhaps just a recombination remnant that has persisted in these genomes by chance.

Another interesting finding was the absence of complete machinery for the *de novo *synthesis of methionine in the *Desulfovibrio *species. These organisms have the necessary genes to form methionine from homocysteine, but no apparent process by which to produce homocysteine. Although the enzymatic pathway of cysteine synthesis has been studied in *Desulfovibrio vulgaris *[53], its ability to synthesize methionine has not been characterized. Growth in minimal medium using sulfate as the only source of sulfur is routine, however, and suggests that these bacteria use a previously uncharacterized mechanism for assimilation of sulfur into methionine. On the basis of genomic context analysis we also predicted that the *Desulfovibrio *species contain a novel set of genes involved in biotin synthesis.

### Regulation of metal-ion homeostasis

A number of regulators believed to be involved in metal-ion homeostasis were identified on the basis of orthology with known factors from *E. coli *or *B. subtilis*. However, in almost all cases, with the possible exception of ZUR and ModE in *G. sulfurreducens*, which appear to have signals similar to the *B. subtilis *and *E. coli *consensus respectively, similarity to known binding signals was not observed (Table 11). The presence of similar sets of target genes in the δ-proteobacteria studied allowed us to apply the signal detection procedure to elucidate novel regulatory signals, to expand core regulons, and to observe species-specific differences in regulation. Interestingly, the FUR/ZUR/PerR family of transcriptional regulators was found to be ubiquitous in these bacteria and responsible for a broad range of functions including iron and zinc homeostasis as well as oxidative stress response. In some cases, multiple paralogous factors were found, perhaps indicating previously uncharacterized functions for this versatile gene family.

The large number of iron-containing proteins predicted from the genome sequence of these organisms, and their ability to use ferric iron anaerobically as a terminal electron acceptor, makes iron homeostasis a key target for analysis. A number of new genes were identified that may belong to the FUR regulon of these organisms. First, uncharacterized porins with upstream FUR boxes were identified in the *Geobacter *and *Desulfuromonas *genomes, which we speculate might be involved in iron transport. Additionally, a two-domain protein with no homologs of known function was identified in all species except *D. psychrophila*. In *G. sulfurreducens*, this gene occurred downstream of another gene with a cytochrome-type heme-binding motif, while in *Desulfuromonas *it was divergently transcribed with a ferric reductase, and was associated with a tetratricopeptide repeat protein in the *Desulfovibrio *genomes. In both *Desulfovibrio *species, we identified an additional regulon, possibly under FoxR control, which might be involved in siderophore transport. This finding was particularly surprising because we did not identify any known siderophore biosynthetic pathway. A possible explanation is that these bacteria use a novel siderophore biosynthesis pathway, or alternatively, take up siderophores released by other bacteria in the environment.

### Stress response

Oxidative stress is one of the most common environmental stressors for these organisms, especially in the metal-contaminated sites of interest for bioremediation. The bacteria in this study are unusual in that they contain both the aerobic superoxide dismutase (Sod)/catalase-type oxidative response as well as the anaerobic Sor/rubrerythrin-type response as previously noted for *D. vulgaris *[54]. Analysis of the signal peptides in these proteins indicates that the Sod/catalase system acts periplasmically, whereas the Sor/rubrerythrin system acts cytoplasmically [54]. While these organisms have no homologs of the OxyR or SoxRS regulators known to respond to changes in oxygen levels in *E. coli*, they do contain homologs of the PerR regulator of *B. subtilis*, known for its involvement in peroxide stress (Table 11). Clustering of PerR homologs with oxidative stress genes, as well as their grouping with known *Bacillus *PerR genes in a phylogenetic analysis of the FUR/ZUR/PerR family of transcription factors, allowed the inference that they may, in part, be responsible for the control of the oxidative stress response of these organisms. Although we did not identify conserved regulatory elements for some known oxidative stress genes such as the Rbo/Rub/Roo operon in *Desulfovibrio *species, it has been observed that the Rub/Roo operon of *Desulfovibrio gigas *shows strong constituitive expression from a previously identified σ^70 ^promoter, indicating that additional factors may not be involved [55].

The heat-shock response of these bacteria was found to be mediated by two regulons previously described in other species (Table 11). First, the σ^32 ^regulon was identified, with a consensus signal similar to that characterized for *E. coli*. The second observed regulon was the HrcA/CIRCE regulon known in *B. subtilis *and other bacteria, but not present in *E. coli*. These two regulons include a partially overlapping set of genes. Notably, CIRCE elements were identified in all of the genomes used in this study with the exception of *D. psychrophila*. It is tempting to speculate that the constant and cold temperatures encountered by this species in its environmental niche have removed the need for this particular heat-shock response.

### Similarity of regulatory signals with those in other bacteria

Comparison with well studied bacterial model organisms has shown that δ-proteobacteria share regulatory components with both Gram-positive and Gram-negative microorganisms (Table 11). For example, the use of NikR and ModE for the regulation of, respectively, nickel and molybdenum uptake and utilization is consistent with *E. coli*-like regulation. However, the presence of PerR, CIRCE elements and S-box motifs is reminiscent of *B. subtilis*-like regulation. Moreover, in the case of FUR, although the regulon structure showed overlap with known downstream targets in model organisms, the sequence of the FUR box, which is conserved in both *E. coli *and *B. subtilis*, was observed to be different in the metal-reducing δ-proteobacteria.

We recognize that this is one of the first direct studies comparing entire regulons in δ-proteobacteria. Two recent computational works, considering either a single *D. vulgaris *or two *Geobacter *species, used the AlignACE signal detection program, which is based on a Gibbs-sampling algorithm, to derive large sets of conserved DNA motifs without linking them to specific regulatory systems [56,57]. Unfortunately, the predicted regulatory signals based on single genomes turned out not to be conserved across genomes, and could not be used for functional gene annotation. In this comparative work, we tried to extensively describe a set of biologically reasonable regulons in δ-proteobacteria. The regulatory sites predicted here were not detected in the other two computational studies by Hemme and Wall and by Yan *et al*. [56,57]. Previously published experimental studies of sulfate-reducing δ-proteobacteria have focused mostly on the biochemistry unique to these organisms, and little is known about the regulation of gene expression. In part, this has been due to difficulties in genetically manipulating these strictly anaerobic bacteria. Recent advances in microarray technologies provide genome-scale expression data for *D. vulgaris *under various conditions. In support of our findings, all operons predicted to be co-regulated by the peroxide-responsive regulator PerR in *D. vulgaris *are significantly downregulated by oxygen stress (J. Zhou, personal communication). Furthermore, recent microarray data obtained for *G. sulfurreducens *in iron-limiting conditions confirm our prediction of the FUR regulon in this genome (R. O'Neil, personal communication).

It is interesting to observe the extent to which regulatory motifs are conserved between δ-proteobacteria. Although riboswitches and some DNA signals (that is, CIRCE, σ^32 ^and BirA) seem to be conserved across vast spans of evolutionary time, in many cases we observe divergence in binding signals even when the core components of a regulon are conserved (NikR, FUR, PerR, ModE). These findings raise, but do not answer, questions such as what circumstances cause transcription factor binding specificities to change or remain conserved, and whether those changes reflect genetic drift, or active selection to alter the regulatory action of the factor.

### Energy metabolism

We identified two regulons involved in the control of energy metabolism (Table 11). The first, controlled by the CooA protein, was present only in the *Desulfovibrio *genomes. It is orthologous to a known regulon in *R. rubrum*, and regulates genes involved in the oxidation of CO. The second regulon is novel and distributed widely among anaerobic and facultatively anaerobic bacteria. The primary downstream target of this newly identified regulator, which we called HcpR*, is the hybrid-cluster protein Hcp. Upregulation of the *hcp *gene in response to growth on nitrate or nitrite in *Shewanella oneidensis*, *E. coli *and *D. vulgaris *indicates that Hcp is likely to be involved in the utilization of alternative electron acceptors.

Consistent with this hypothesis, we predicted positive regulation of Hcp and the associated ferredoxin FrdX by HcpR, and negative regulation of the sulfate-reduction genes by HcpR in the *Desulfovibrio *genomes, based on the position of the candidate HcpR-binding sites relative to the predicted promoters. Thus, HcpR is predicted to be responsible for switching between alternative electron acceptors during anaerobic respiration in these species. Interestingly, we found an HcpR site upstream of the CO-dependent hydrogenase that was also predicted to be under the control of CooA. This hydrogenase was recently proposed to play a key role in sulfate reduction [16], and it is tempting to speculate that its inclusion in a common regulon with known sulfate-reduction genes supports this hypothesis. The position of the binding site, however, suggests that it activates rather than represses transcription, contrary to predictions for other known sulfate-reduction genes, so its regulation is likely to be complex, and further experiments will be needed to determine whether it plays the role of the cytoplasmic hydrogenase necessary for the proposed 'hydrogen cycling' of sulfate reduction [58]. The ubiquitous phylogenetic distribution of the HcpR regulon indicates that it has a central role in facilitating an anaerobic life style, yet very little is known about its specific function. We hope our elucidation of the core components and regulator of this important regulon will inspire future experimental studies to determine its cellular role.

### Regulatory motifs for alternative cofactor adaptation

In the course of this study we identified several cases in which different variants of genes were predicted to be regulated according to the availability of required cofactors or nutrients. Three examples were observed in which an alternative enzyme, not requiring a given cofactor, was repressed by the availability of that cofactor: B_12_-independent ribonucleotide reductase was repressed by the availability of B_12_; [Fe] hydrogenase was repressed by the availability of nickel (and presumably replaced by [NiFe] hydrogenase); and Fe(II) was predicted to repress a flavodoxin gene which we suspect may be used as an alternative to ferredoxins present in the genome. This mode of regulation for B_12_-independent isozymes of ribonucleotide reductase and methionine synthetase has been previously described [26]. Moreover, a similar regulatory strategy has been reported for one of the alternative superoxide dismutases and for paralogs of ribosomal proteins [34-36,38,59]. Taken together, these data suggest that this flexible strategy may represent a common theme in the adaptation of bacteria to their environment. Indeed, similar mechanisms may, in part, explain some of the apparent genetic redundancy in many genomes.

## Materials and methods

The genomes of δ-proteobacteria that were analyzed in this study are *Desulfovibrio vulgaris *Hildenborough (DV); *Desulfovibrio desulfuricans G20 *(DD); *Geobacter metallireducens *(GM); *Geobacter sulfurreducens PCA *(GS); *Desulfuromonas *species (DA); and *Desulfotalea psychrophila *(DP). Complete genomic sequences of DV and GS were downloaded from GenBank [60]. Draft sequences of DD, GM and DA genomes were produced by the US Department of Energy Joint Genome Institute and obtained from [61]. Draft sequence of the DP genome was provided by the Max Planck Institute for Marine Microbiology in Bremen, Germany [62]. Numerical gene identifiers from the Virtual Institute for Microbial Stress and Survival (VIMSS) Comparative Genomics database [63] are used for hypothetical genes without common names. New gene names introduced in this study are marked by an asterisk.

For *de novo *definition of a common transcription factor-binding signal in a set of upstream gene fragments, a simple iterative procedure implemented in the program SignalX was used [31]. Weak palindromes were selected in each region, and each palindrome was compared to all others. The palindromes most similar to the initial one were used to make a profile. The positional nucleotide weights in this profile were defined as

*W*(*b,k*) = log[*N*(*b*,*k*) + 0.5] - 0.25Σ_i = A,C,G,T_log[*N*(*i*,*k*) + 0.5],

where *N*(*b*,*k*) is the count of nucleotide *b *in position *k *[10]. The candidate site score *Z *is defined as the sum of the respective positional nucleotide weights

*Z*(*b*_1_...*b*_*L*_) = Σ_*k *= 1...*L*_*W*(*b*_*k*_,*k*),

where *k *is the length of the site.

These profiles were used to scan the set of palindromes again, and the procedure was iterated until convergence. Thus a set of profiles was constructed. The profile with the greatest information content [64] was selected as the recognition rule.

Each genome was scanned with the profile using the GenomeExplorer software [65], and genes with candidate regulatory sites in the 300-bp upstream regions were selected. The upstream regions of genes that are orthologous to genes containing regulatory sites were examined for candidate sites even if these were not detected automatically. The threshold for the site search was defined as the lowest score observed in the training set. Sets of potentially co-regulated genes contained genes that had candidate regulatory sites in their upstream regions and genes that could form operons with such genes (that is, located downstream on the same strand with intergenic distances of less than about 100 bp). A complete description of the GenomeExplorer software, including the SignalX program, is given at [65].

The RNApattern program [66] was used to search for conserved RNA regulatory elements (riboswitches) in bacterial genomes. The input RNA pattern for this program describes an RNA secondary structure and sequence consensus motifs as a set of the following parameters: the number of helices, the length of each helix, the loop lengths, and a description of the topology of helix pairs. The latter is defined by the coordinates of helices. For instance, two helices may be either independent or embedded helices, or they could form a pseudoknot structure. This definition is similar to the approach implemented in the Palingol algorithm [67].

Orthologous proteins were identified as bidirectional best hits [68] by comparing the complete sets of protein sequences from the two species using the Smith-Waterman algorithm implemented in the GenomeExplorerprogram [65]. When necessary, orthologs were confirmed by construction of phylogenetic trees for the corresponding protein families. Phylogenetic analysis was carried out using the maximum likelihood method implemented in PHYLIP [69]. Large-scale gene cluster comparisons were carried out using the VIMSS Comparative Genomics database [63]. Multiple sequence alignments were done using CLUSTALX [70]. The COG [68], InterPro [71], and PFAM [72] databases were used to verify the protein functional and structural annotation.

### Note added in proof

Recently it has been demonstrated by *in vitro *experiment that the glycine-specific riboswitch consists of two tandem aptamer sequences that appear to bind target molecules cooperatively [73]. This indirectly confirms our hypothesis of a cooperative effect of ligand binding to tandem *THI*-elements in *Desulfovibrio *spp. Also we have recently shown that *Geobacter *spp. have a modified HcpR regulon, which uses a signal similar to that found in DA and DP, but contains multiple nitrate/nitrite reductase genes.

## Additional data files

An additional data file (Additional data 1) containing three figures with detailed description of DNA- and RNA-type regulatory sites is available with the online version of this paper and on our website [74].

## Supplementary Material

Additional data file 1 Three figures with detailed description of DNA- and RNA-type regulatory sites Click here for additional data file

## Figures and Tables

**Table 1:** Candidate binding sites for the biotin repressor BirA

Gene		Site	Position*	Score
*Desulfuromonas *sp.				
387978	*bioW*	aTGTcAACC-[N_14_]-GGTTgACAg	-63	8.61
390011	*bioB*	acGTcAACC-[N_14_]-GGTTgACAA	-94	8.13
				
*Geobacter sulfurreducens *PCA				
381880	*bioB*	TTGTcAACC-[N_14_]-aGTTgACAA	-78	8.50
382941	*bioF*	TTGTcAACC-[N_14_]-GGTTgACgA	-182	8.29
				
*Geobacter metallireducens*				
377241	*bioB*	TTGTtAACC-[N_14_]-aGTTgACAA	-76	7.81
377542	*bioF*	TTGTcAACC-[N_14_]-GGTTgACgA	-64	8.29
				
*Desulfovibrio vulgaris*				
208055	*bioB*	TTGTAAACC-[N_15_]-cGTTgACAg	6	8.39
				
*Desulfovibrio desulfuricans *G20				
394249	*bioB*	TTGTAAACC-[N_15_]-aGTTgACAA	-119	8.60
				
*Desulfotalea psychrophila*				
425025	*bioB*	TTGTAAAtt-[N_15_]-ccaTTACAg	233	6.19

*Position relative to the start of translation. Lower case letters represent positions that do not conform to the consensus sequence.

**Table 2:** Candidate binding sites for the predicted lysine-specific regulator LysX*

Gene		Site	Position^†^	Score
*Desulfovibrio vulgaris*				
208064	lysX*-lysA	GTGGTACTAATcAGTACCAC	-277	6.82
206613	~mviN*	GTGGTtCTttgTAGTACtAC	-135	5.45
				
*Desulfovibrio desulfuricans *G20				
394240	lysX*-lysA	GTaGTACTAAaTAGTACCAC	-43	6.70
393213	lysW*	GgcGTtCTAAagAGTACCAC	-145	5.88
394397	~mviN*	GTaGTtgTgATaAGaAaCAC	-275	4.70

^†^Position relative to the start of translation. *New name introduced in this study. Lower case letters represent positions that do not conform to the consensus sequence.

**Table 3:** Candidate binding sites for the ferric uptake regulator FUR

Gene	Operon	Function	Site	Position*	Score
*Geobacter sulfurreducens *PCA					
381665	*Fur*	Ferric uptake regulator	ATGAtAtTCAcTTTCAg	-31	5.25
381666	*feoB1 - R*	Fe^2+ ^transporter	cTGAAAgTGATTTTCAc	-192	5.18
383594	genX*-genY*	Cytochrome c family protein, putative	gTGAAAAaCATTTTCAa	-65	5.08
383590	*X-feoA-feoA-feoB2*	Porin, Fe^2+ ^transporter	tTGAAAATGgaaTTCAT	-82	5.07
					
*Geobacter metallireducens*					
379927	*Fur*	Ferric uptake regulator	tTGAAAATCAcTTTCAg	-30	5.54
379928	*feoB1 - R*	Fe^2+ ^transporter	tTGAAAgTGAaTaTCAa	-48	5.33
378774	psp*	Porin?	**tTGAAAAaGAcTTTCAT**	**-259**	5.28
			**ATGAAtATGAaTTTCAa**	**-160**	5.35
					
*Desulfuromonas *species					
392427	*fur2-feoB1 - R*	Fe regulator, Fe^2+ ^transporter	tTGAAAATCATTTTCAg	-34	5.72
390939	psp*	Porin?	**tTGAtAATGgcTTTCAT**	**-139**	5.22
			**cTGAAAAcGATTTTCAT**	**-86**	5.46
391943	*fur1*	Ferric uptake regulator	tTGAAcATCATTTTCAT	-37	5.44
387887	*feoA-feoB4*	Fe^2+ ^transporter	ATGAAAAcGAaTTTCAT	93	5.43
			tTGAtAAaGAcTTTCAT	39	5.12
391875	genY*(N)		tTGAAAAcGgTTTTCAT	-105	5.28
389803	*feoA-feoB2*	Fe^2+ ^transporter	cTGAAAAcCgTTTTCAa	-39	5.16
392265	*feoA-feoB3*	Fe^2+ ^transporter	ATGAAAtaCAcTTTCAa	-54	5.13
					
*Desulfovibrio vulgaris*					
209207		?	**tTGAAAATtATTTTCAa**	**-35**	5.42
			**ATtAtttTCAaTaTCAg**	**-29**	4.06
206189	gdp*	GGDEF domain protein	**tTGActtTGAaaaTCAT**	**-36**	4.04
			**tTGAAAATCATaaTCAa**	**-30**	5.32
208071	*feoA-feoA-feoB*	Fe^2+ ^transporter	**ATaAActTGAcaaTCAT**	**-99**	3.91
			**tTGAcAATCATTTTCAT**	**-93**	5.18
207866	foxR-pqqL*-atpX*-...	Regulator, Zn-dependent peptidase, ABC operon	**tTGActtTGATTTTCAc**	**-195**	4.31
			**tTGAtttTCAcTTTCAT**	**-189**	5.01
209238	genY*(C)-genZ*	?	**tTGAcAtTGATTTTCgT**	**-55**	4.31
			**tTGAtttTCgTTTTCAa**	**-49**	4.89
208179	fld*	Flavodoxin	**tTGAAAAcaAaaaTCAa**	**-182**	4.49
			**AcaAAAATCAaTTTCAa**	**-176**	4.25
208641	hdd*	HD-domain protein	**tTGAcAATGATTTTCtT**	**-93**	4.46
			**ATGAtttTCtTTTTCAa**	**-87**	4.81
208856		Has P-type ATPase/hydrolase domains	**tTGAtttaGATTTTCAa**	**-87**	4.79
			**taGAtttTCAaTTTCAg**	**-81**	4.20
			**tTcAAttTCAgTaTCAa**	**-75**	3.82
					
*Desulfovibrio desulfuricans *G20					
395878	*fur3*	Ferric uptake regulator	ATGAAAATaATTTTCAT	-77	5.46
393004	pqqL*-atpX*-...	Zn-dependent peptidase, ABC operon	**ATGAAAATaAaTTTCAT**	**-54**	5.31
			**ATaAAttTCATTTTCAT**	**-48**	4.65
392971	*392971-70-69*	MoxR-like ATPase, CoxE-like protein	**cTGAAAtTGgTTTTCAa**	**-99**	5.29
			**tTGgtttTCAaTaTCAg**	**-93**	4.24
			**tTGAAAATGAaaTTtAT**	**-30**	4.63
			**ATGAAAtTtATagTCAg**	**-24**	4.19
393146	genY*(C)-genZ*	?	**tTGAcAtTGATTTTCAT**	**-84**	5.03
			**tTGAtttTCATTTTCAc**	**-78**	4.81
393462	fld*	Flavodoxin	**tTGAcAATGAaTTTCAT**	**-263**	5.03
			**ATGAAttTCATTTTCAc**	**-257**	4.99
394236	*feoA-feoB*	Fe^2+ ^transporter	**ATGAgAAgGATTTTCAa**	**-83**	5.00
			**AgGAtttTCAaTTTCAc**	**-77**	3.96
394235	*feoA3*	Fe^2+ ^transporter	**AgGAActTGAcaaTCAT**	**-60**	3.91
			**tTGAcAATCATTcTCAT**	**-54**	4.72
393956	gdp*	GGDEF domain protein	**tTGAtttTGAgTTTCAT**	**-122**	4.56
			**tTGAgttTCATaTTCAT**	**-116**	4.55
395154	*FoxR*	AraC-type regulator	**tTGAcAtTGAaaaTCAT**	**-189**	4.38
			**tTGAAAATCATTTTCgc**	**-183**	4.74
394231	pep*-fur1	Zn-dependent peptidase, Fe regulator	**tTcAgAcTGgTTTTCAT**	**-281**	3.75
			**cTGgtttTCATTaTCAT**	**-275**	4.41
395541	hdd*	HD-domain protein	**gTGAtAtTGAaaTTCtT**	**-105**	3.96
			**tTGAAAtTCtTTaTCgc**	**-99**	4.05
395164	*fepA-feoA2-feoB2*	Outer membrane receptor, Fe-transporter	**cTGAtAAaGAaacTCAc**	**105**	3.87
			**AaGAAAcTCAcTaTCAg**	**111**	4.05

*Position relative to the start of translation. Lower case letters represent positions that do not conform to the consensus sequence. Multiple tandem sites in one regulatory region are shown in bold.

**Table 4:** Candidate binding sites for the nickel regulator NikR

Gene	Operon	Function	Site	Orientation	Position*	Score
*Geobacter sulfurreducens *PCA						
381565	nik(MN)QO*	Nickel transporter	GTGTTAC-[N_14_]-GTgACAC	→←	-183	5.00
						
*Geobacter metallireducens*						
379930	nik(MN)QO*	Nickel transporter	GTGTTAC-[N_13_]-GTAACAC	→←	-63	5.22
						
*Desulfuromonas *species						
387207	nikQO*	Nickel transporter	GTGccAC-[N_13_]-GTAACAC	→←	-41	4.67
						
*Desulfovibrio vulgaris*						
206492	nikMQO*	Nickel transporter	GTGTTAt-[N_13_]-GTAACAC	→←	-120	5.00
208275	nikK*	Additional component of Ni transporter	GTgACAC-[N_13_]-GTGTaAC	←→	-84	4.49
						
*Desulfovibrio desulfuricans*						
395510	nikKMLQO*	Nickel transporter	GTGTTAt-[N_13_]-GTAACAC	→←	-104	5.00
394565	*hydAB*	Periplasmic Fe-only hydrogenase	GTaTTAC-[N_13_]-GTAACAC	→←	-83	4.67
						
*Desulfotalea psychrophila*						
422915	nikMLKQO*	Nickel transporter	GTAACAC-[N_13_]-GTGTTAC	←→	-20	5.22
422176	422176-177	?	GTAACAC-[N_13_]-GTGTTAC	←→	-197	5.22
			GTAACAC-[N_13_]-GTGTTAC	←→	-124	5.22

*Position relative to the start of translation.

**Table 5:** Candidate binding sites for the zinc regulator ZUR

Gene	Operon	Function	Site	Position*	Score
*Geobacter sulfurreducens *PCA					
383303	*zur_Gs-znuABC*	Zinc ABC transporter, regulator	TAAAtgGAAATgATTTCtgTTTA	-40	5.32
					
*Desulfovibrio vulgaris*					
206785	*znuABC-zur_D*	Zinc ABC transporter, regulator	ATGCAACagtGTTGCAT	-216	6.65
					
*Desulfovibrio desulfuricans*					
394629	*znuABC-zur_D*	Zinc ABC transporter, regulator	ATGCAACtgaGTTGCAT	-47	6.65

*Position relative to the start of translation. Lower case letters represent positions that do not conform to the consensus sequence.

**Table 6:** Candidate binding sites for the molybdate regulator ModE

Gene	Operon	Function	Site	Position*	Score
*Geobacter sulfurreducens PCA*					
383279	*modDABC*	Molybdate transport	ATCGTTATgTcaTgAAggtTATAGCGtT	-158	5.16
					
*Desulfovibrio vulgaris*					
209110	*modA*	Molybdate transport	CGGTCACG-[N_14_]-gGTGACCG	-131	5.56
209114	*modBC*	Molybdate transport	CGGTCACc-[N_14_]-CGTGACCa	-218	5.38
					
*Desulfovibrio desulfuricans*					
393254	*modAB2-393256*	Molybdate transport, ?	CtGTCACG-[N_14_]-CGTGACCG	-183	5.56
393587	*modAB1-modC*	Molybdate transport	ttGTCACG-[N_14_]-CGTGACCG	-119	5.38

*Positionrelative to the start of translation. Lower case letters represent positions that do not conform to the consensus sequence.

**Table 7:** Candidate binding sites for the peroxide-responsive regulators PerR

Gene	Operon	Function	Site	Position*	Score
*Desulfovibrio vulgaris*					
207805	*rbr2*	Rubrerythrin	AATAGGAATCGTTCCTGTT	-46	5.97
208612	*perR-rbr-rdl*	PerR-like repressor, rubrerythrin, rubredoxin	AtCAGTAATtGTTACTGgT	-36	5.50
207732	*ahpC*	Alkyl hydroperoxide reductase C	cACAGGAATGATTCCTGTT	-116	5.40
206199		?	AtCAGTAATaGTTAtTGTT	-124	5.39
					
*Desulfovibrio desulfuricans*					
395420	*rbr2*	Rubrerythrin	AATAGGAATCGTTACTGaT	-76	5.91
395549		?	AATAaGAATtGTTACTATT	-134	5.45
393457	*perR*	PerR-like repressor	ttTAGGAATGGTTAtTATT	-41	5.23
					
*Desulfotalea psychrophila*					
423938	*roo1-roo2*	Rubredoxin-oxygen oxidoreductase	GTTAATGATAATCATTAct	-203	6.25
425393	*perR*	PerR-like repressor	GaTAATttTTATtATTAAC	-74	5.97
					
*Geobacter sulfurreducens*					
383613	perR-rbr*-roo	Rubredoxin-oxygen oxidoreductase	AaTGCAATAAAATACCAAT	-99	6
					
*Geobacter metallireducens*					
378323	perR2-rbr*-roo	Rubredoxin-oxygen oxidoreductase	ATTGCAATAAAgTACCAAc	-99	5.79
					
*Desulfuromonas *species					
387528	*katG1*	Catalase	GGTcTTGACAATtCC	-75	5.55
387530	*perR31*	PerR-like repressor	GaTATTGACAAacCC	-96	5.29
					
*Geobacter sulfurreducens*					
383124	*hsc-grx-ccpA-rbr*	Cytochrome peroxidase, glutaredoxin, rubrerythrin	TTGCGCATTCcATtCGTAA	-32	5.84
					
*Desulfuromonas *species					
390120	*perR1-rbr*	PerR-like repressor, rubrerythrin	TTGCGCgTTAAAacaGTAA	-91	5.54

*Position relative to the start of translation. Lower case letters represent positions that do not conform to the consensus sequence.

**Table 8:** Candidate CIRCE sites for the heat shock-responsive regulator HrcA

Gene	Operon	Site	Position*	Score
*Desulfovibrio vulgaris*				
207448	*groESL*	cTgGCACTC-[N_9_]-GAGTGCcAA	-68	6.53
				
*Desulfovibrio desulfuricans*				
394393	*groESL*	TTgGCACTC-[N_9_]-GAGTGCTAA	-70	7.15
				
*Geobacter sulfurreducens*				
380317	*hrcA-grpE-dnaK-dnaJ*	TTAGCACTC-[N_9_]-GAGTGCTAA	-49	7.50
380945	*rpoH*	TTAGCACTC-[N_9_]-GAGTGCTAA	-51	7.28
383663	*groESL*	TTAGCACTC-[N_9_]-GAGTGCTAA	-81	7.45
				
*Geobacter metallireducens*				
379288	*groESL*	TTAGCACTC-[N_9_]-GAGTGCTAA	-80	7.41
379629	*hrcA-grpE-dnaK-dnaJ*	TTAGCACTC-[N_9_]-GAGTGCTAA	-45	7.29
				
*Desulfuromonas *species				
387711	*hrcA-grpE-dnaK-dnaJ*	TTAGCACTC-[N_9_]-GAGTGCTAA	-85	7.06
389722	*groESL*	TTAGCACTC-[N_9_]-GAGTGCTAA	-99	7.20

*Position relative to the start of translation. Lower case letters represent positions that do not conform to the consensus sequence.

**Table 9:** Candidate σ^32^-dependent promoters upstream of heat-shock genes

Gene	Operon	Site	Position*	Score
*Desulfovibrio vulgaris*				
206437	*dnaJ-?-clpA*	gaTGAAt-[N_15_]-CCCCtT	-114	5.43
206776	*?-clp*	gTTGttg-[N_15_]-CCCCgT	-196	5.28
207035	*rpoH*	aTTGAAA-[N_12_]-aaCtAT	-110	5.71
207448	*groESL*	CaTaAAA-[N_12_]-CCCCtT	-239	5.23
				
*Desulfovibrio desulfuricans*				
394616	*clpP-clpX-lon*	CTTGAAc-[N_12_]-CCCgAT	-82	6.45
394617	*clpX*	CTTGAAA-[N_14_]-aCCgAT	-136	6.94
394712	*rpoH*	aTTGAAA-[N_12_]-aaCtAT	-122	5.71
395109	*dnaJ-?-clpA*	CTTGAAA-[N_13_]-gaCggT	-81	5.16
		gTTGcAg-[N_12_]-CCgCAT	-57	5.28
395651	*dnaK*	CTcGAAA-[N_14_]-CCgCAg	-71	5.17
				
*Desulfotalea psychrophila*				
422219	*groESL*	aTTGAAA-[N_13_]-CCCCtT	-201	6.33
		CTTGAtt-[N_13_]-aCCtAT	-134	5.98
423932	*grpE-dnaK*	CaTGAAc-[N_12_]-CtCCAT	-232	5.34
		CTTGAcA-[N_13_]-aCttAT	-135	5.67
424328	*dnaJ*	gTTtAcA-[N_14_]-gCCCAT	-113	5.62
		CTTGAct-[N_14_]-CCCtAa	-40	5.67
425016	*?-clpP-clpX-lon*	tTTGAtA-[N_11_]-CCCaAg	-123	5.33
				
*Geobacter sulfurreducens*				
380319	*dnaK-dnaJ*	gTTGAgg-[N_14_]-CCCaAT	-208	6.05
382089	*?-clpP-clpX-lon*	gTTcAAA-[N_12_]-CCCCAT	-283	6.65
382697	*htpG*	CTTGAAA-[N_11_]-CatgAT	-75	5.85
				
*Geobacter metallireducens*				
379288	*groESL*	gaTGAAA-[N_12_]-aCtCAT	-45	5.79
379647	*clpA*	CTTGAct-[N_14_]-gCCtAT	-58	5.72
379699	*?-clpP-clpX-lon*	gTTcAAA-[N_13_]-CCCaAT	-280	5.96
				
*Desulfuromonas *species				
388073	*clpP-clpX-lon*	CTTGAAg-[N_14_]-gCCaAT	-203	6.41
		aTTGAAg-[N_14_]-aCCtAT	-110	6.20
389722	*groESL*	gTTGAgA-[N_14_]-CCCCtT	-163	5.91

*Position relative to the start of translation. Lower case letters represent positions that do not conform to the consensus sequence.

**Table 10:** Candidate binding sites for the CO-responsive regulator CooA and the FNR/CRP-like HcpR factor

Gene	Operon	Function	Site	Position*	Score
**(a) **CooA regulon					
*Desulfovibrio vulgaris*					
207573	*cooSC*	CO dehydrogenase (CODH)	TGTCGGCTAGCCGACA	-187	6.04
207772	*cooMKLXUHXF*	CODH-associated hydrogenase	gGTCGGtcAaCCaACt	-64	4.43
				
*Desulfovibrio desulfuricans*					
393975	*cooSC*	CO dehydrogenase (CODH)	TGTCaGCcAGCCGACA	-111	5.78
				
**(b) **HcpR regulon					
*Desulfovibrio vulgaris*					
208467		Two-component response regulator	TTGTGAcATgTaTaACAA	-74	5.61
206736	*sat*	ATP sulfurylase	TTGTaAAtTtTTTCACAA	-148	5.53
206272	*apsAB*	APS reductase	TTGTtAAtTccaTCACAA	-168	5.29
209106	*phcAB*	Putative thiosulfate reductase	aTGTGAcgcATTTCgCAA	-194	5.06
207772	*cooMKLXUHXF*	CODH-associated hydrogenase	TTGgGAAtcgaTTCACAA	-116	4.97
208738	208738-208737	Two-component regulatory system	cTGTGAAAcATgTCgCAt	-104	4.88
206515	206515-206516	Putative sulfite/nitrite reductase, polyferredoxin	gTGTGAcccgcgTCACAg	-52	4.79
209119		Hypothetical protein conserved in Archaea	TTGTtcAcaAaaTCACAA	-218	4.61
208040	*hcp-frdX-adhE*-208043	Hybrid cluster-containing protein, ferredoxin, alcohol dehydrogenase, histidine kinase	aTtTGAcgcAcgTCACAA	-179	4.55
				
*Desulfovibrio desulfuricans*					
392869	209119	Hypothetical protein conserved in Archaea	TTGTtAAATAaTTCACAA	-118	5.93
395578	*apsAB*	APS reductase	TTGTtAAATATcTCACAA	-186	5.77
394579	*sat*	ATP sulfurylase	TTGctAAAaATTTCACAA	-147	5.43
			TTGTtAcAatTaTCACAt	-328	4.93
393955		Two-component response regulator	TTGTGAcAgcTgTCACAA	-80	5.36
393201		Two-component response regulator	TTGTGAAggAaaTaACAA	-18	5.29
392939		~ 6-aminohexanoate-cyclic-dimer hydrolase	TTGTtAAtTATTTaAaAA	-61	5.00
395499	395499-395498-395497-395496	Arylsulfatase, thioredoxin, thioredoxin reductase, sulfate transporter homolog	aTGTGAAAaAcaTCACAt	-129	4.98
393758	393758-..-393776	Large gene cluster encoding carboxysome shell proteins, aldehyde dehydrogeanses, ...	TTGTtAtATtTTTCtCAA	-148	4.97
394469	394469-394470	Putative sulfite/nitrite reductase, polyferredoxin	aTGTGAccTgcaTCACAg	-81	4.86
394261	*hcp-frdX-uspA*	Hybrid cluster-containing protein, ferredoxin, universal stress protein UshA	TTGTGActccggTCACAt	-152	4.81
395604	*phcAB*	Putative thiosulfate reductase	TTGTGcttTtTTgCACAA	-114	4.25
				
*Desulfotalea psychrophila*					
425344	*frdX*	Ferredoxin	ATTTGAtCTAGGTCAAAg	-103	5.81
423439	*hcp3/hcp2*	Hybrid cluster-containing proteins	ccTTGACCTgGGTCAAtT	-200	5.47
422894	*hcp1*	Hybrid cluster-containing protein	tcTTGACtTAGGTCAAAg	-117	5.44
				
*Desulfuromonas *species					
389812	*hcp1/?-frdX2-?*	Hybrid cluster-containing protein/ferredoxin	ATTTGACCTcGGTCAAga	-155	5.66
			AcaTGACgcAGaTCAAAa	-200	4.87
389024	*hcp3*	Hybrid cluster-containing protein	tcTTGAtCTgGaTCAAAT	-85	5.45
391271	*dnrA*	~ Regulator of NO signaling	cTTTGACCcgGGTCAAtT	-109	5.44
390920	*hcp2*	Hybrid cluster-containing protein	ATTTGACCTgGGTCAtgT	-127	5.40
390344	*galE*	~ Nucleoside-diphosphate-sugar epimerase	ATTTGACCccGGTCAAta	-117	5.39
392163	*yccM*	Polyferredoxin	AaaTGACCcAGGTCAAAg	-80	5.14
392663		Two-component response regulator	AaTTGAttcAGGTCAAgg	-85	5.06
390999		Cytochrome c (heme-binding protein)	ATTTGACggccGTCAAAg	-83	5.02
390998	*frdX1*	Ferredoxin	tTTTGAtgccGGTCAAgg	-96	5.00
388470	*hcp4*	Hybrid cluster-containing protein	tTTTGAttTgtaTCAAtT	-126	4.66

*Position relative to the start of translation. **(a) **Candidate sites of the CO-responsive regulator CooA in *Desulfovibrio *species; **(b) **candidate sites of the FNR/CRP-like HcpR factor regulating energy metabolism. Lower case letters represent positions that do not conform to the consensus sequence.

**Table 11:** Summary of predicted regulatory sites in δ-proteobacteria

Regulator	Regulon	Consensus	Genomes
BirA	Biotin biosynthesis	TTGTAAACC-[N_14/15_]-GGTTTACAA	DD, DV, GM, GS, DA, DP
*RFN *riboswitch	Riboflavin biosynthesis	see Additional data files	DP
*THI *riboswitch	Thiamin biosynthesis	see Additional data files	DD, DV, GM, GS, DA, DP
*B*12 riboswitch	Cobalamin biosynthsis and transport	see Additional data files	DD, DV, GM, GS, DA, DP
S-box riboswitch	Methionine biosynthesis	see Additional data files	GM, GS, DA
LysX	Lysine biosynthesis and transport	GTgGTaCTnnnnAGTACCAC	DD, DV
Fur	Iron uptake and metabolism	GATAATGATnATCATTATC	DD, DV, GM, GS, DA
NikR	Nickel uptake and metabolism	GTGTTAC-[N_13/14_]-GTAACAC	DD, DV, GM, GS, DA, DP
Zur	Zinc uptake	ATGCAACnnnGTTGCAT	DD, DV
		TAAATCGTAATnATTACGATTTA	GS
ModE	Molybdate uptake and metabolism	cgGTCACg-[N_14_]-cGTGACCg	DD, DV
		atCGnTATATA-[N_6_]-TATATAnCGat	GS
PerR	Peroxide stress response	AwnAGnAAtngTTnCTnwT	DD, DV
		TtnCgnnTTnAAnncGnAA	DA, GS
		AatTGnnATnnnATnnCAatt	GM, GS-2
		GtTAATgATnATcATTAaC	DP
		GgnnTTGnCAAnncC	DA-2
HrcA	Heat-shock response	TTAGCACTC-[N_9_]-GAGTGCTAA	DD, DV, GM, GS, DA
Sigma-32	Heat-shock response	CTTGAAA-[N_11/16_]-CCCCAT	DD, DV, GM, GS, DA, DP
CooA	CO dehydrogenase	TGTCGGCnnGCCGACA	DD, DV
HcpR	Sulfate reduction and energy metabolism (prismanes)	TTGTGAnnnnnnTCACAA	DD, DV
		atTTGAccnnggTCAAat	DA, DP

DV (*Desulfovibrio vulgaris*); DD (*Desulfovibrio desulfuricans G20*); GM (*Geobacter metallireducens*); GS (*Geobacter sulfurreducens PCA*); DA (*Desulfuromonas *species); DP (*Desulfotalea psychrophila*). Lower case letters represent positions that do not conform to the consensus sequence.
