# Supplementary material for: Reconstruction of regulatory and metabolic pathways in metal-reducing δ-proteobacteria
Source: Genome Biol. 2004 Oct 22;5(11):R90. doi: 10.1186/gb-2004-5-11-r90 (PMC545781; doi:10.1186/gb-2004-5-11-r90)

DESCRIPTION OF ADDITIONAL DATA FILES

Supplementary Fig. 1. (A) The thiamin-specific regulatory *THI* elements in δ-proteobacteria. *THI*-elements are in bold with conserved sequence regions in red and conserved helices marked by background gray, yellow, green and blue colors. Regulatory hairpins (terminators) are marked by background magenta with poly-T regions in blue. Start codons and ribosome-binding sites are in blue and underlined.

DD_THIS
CAGCTAGGGGTGCCCGTACGGGCTGAGATTGAAACAGGTGTTTCTGACCCTTGGAACCTGATGCGGGTAACCGCCGTAGGGAAGCTGGCAGACATATCATACATATGCAGCCGGCCTGAGCC
GGCTTTTTTTGTATGTATGACCAGGGCGCGTATGCGCCAGTGCTGGGGGGGCCTTGTCAGGCTGAGATTGAAGCGCAAAGCTTCTGACCCCTTGAACCTGATGCAGTTAACACTGCCGAAGG
GAAGCCATGATATTTTCATTGCGGAAATGCTGCGGCTTGTCTTTCGGGCAGGCCGCTTTTTTTATGTCCGATGTATGAAAGACAGCAGCGCGCCGCCCGCCGGAAGATTCCGGCGTTGCCGG
ACGCTGCGGCCTGTCGGGTGCAGAACAAAAGGCGGAACAATGAACATCA
DV_THIS
TCAGCTAGGGGAGCCTTCGGGCTGAGAGTGGGCACGTCCCAGACCCTGTGAACCTGACGCAGTTCACACTGCCGTAGGGAAGCTGAGCGCCGGACAACGTCCGTTCGCCCATCCTCTGCGGA
TGGGCTTTTTTGTTTTTCGAGTATCAGCGGTCGCCGTCGCGACCGGAGCTAGGGGAGCCTTCGGGCTGAGAGTGGGTTCGTCCCAGACCCTGTGAACCTGACGCAGTTAGCACTGCCGTAGG
GAAGCCGCGCACCGGACATGCGTCCGTACGCCCATCCTCTGCGGATGGGCGTTCTGTTTTTCCGGCGCCCGCCGCATCGCCTTTGCGCCCTGCCTGCGACTATGGCGGCAGGGCAGGCGGGG
CGGTCGGCCTTGGCAGGCGTCAGCAGACAACCCGGCAGAAGGAGAGACGGCGATGACGATAG
DA_THIF
GCCCTGGGGGAGTTTATTACTGAGAGTCCTGTTGCGGGACGACCCTTAGAACCTGATCCGGGTTATACCGGCGTAGGGAAGCGGCACTTTATACGGGATTTCTCCCGTCGAAGGTCGCTTTC
GCGACCTTTTTCTATTTTGGATCCAAGAGCACTTTCCGCTCACCAATGTGTCATGATTATCG
DA_THIE
ATGGCTGCTGGGGGAGTGATTCACTGAGAGCTCTGTTAGATTGGAGCGACCCTTGGAACCTGATCCGGATTATACCGGCGTAGGGAAGCGACCTGACAACACCGTATGGTGTTCG **direct sequestration of the ribosome-binding site (regulation of translation)**
GM_THIE
TTGACTTAACGGGAAAATTCGGTTAGCTTTGTCTACACAAAAGCAGCCTGCTGGGGGAGTTCTTCGAGAACTGAGACGGGCACCGCCCGAACCCTTACCACCTGATCCGGGTAATGCCGGCG
TAGGGAAGCGGCCAGAGACCTCGTTTACCTCGTTGAGTTCTGGGACCGTGACCATCGTCACGGTTTTTTTGTTTGGGAGATCACCATGGGCAGCA
GM_THIS
CGCTAGGGGAGTTTCGACTGAGAGTCCGACGCGCTGCTACGGACAACCCTTTGAACCTGATCCGGATAATGCCGGCGTAGGGAAGCGTTCCACCATGGTCTATTCCACGACCACGGGCCGCT
TCTGCGGCCTTTTTTGTTTACGCGACCAGATGCCGGTTTCGACGAACCGGCACCCATACTCGGAGAACACAATGGAAATCA
GS_THIE
CTGCTGGGGGAGTTCTTGGGAACTGAGACGGGCAACGCCCGAACCCTTTGAACCTGATCCGGTTTATACCGGCGTAGGGAAGCGGCCAGAAACAATCATCCGTACGTCTTTTCTGGAACCGC
GACTTTGCCGTCGCGGTTTTTTTTTGAGGAGATTTCCTTTCATGGCCTCT
GS_THIS
CGCTAGGGGAGTTTCGGCTGAGAGCCCGTCTGTGATGCCGTTCGGACCGCGCCCGGATAACAGGGAATAACGCGGCACGGAGCGGCAGGGCGACCCTTCGAACCTGATCCGGGTAATACCGG
CGTAGGGAAGCGATAGAACATGGATCACCACAACCCATGGGCCGCTTCTGCGGCCTTTTTTGTTTACACAACCCCACCGTATCCCGACAGGAGGCCCCATGAAC
DP_THIM
TTTTTGCAGGGGTGGCTTTAGGGCCTGAGAAGATACCCATTGAACCTGACCTGGCTAAAACCAGGGTAGGGAATTGCAGAAATGTCCTCATTTTCATTTCTTCGCTCTATCTGCTCGCCCTC
CCCCTGTTAATTCTAGCGAAAGCTTTAACATTTATCCGCTGAGTAATGTTATGTCGA

(B) Conserved structure of the *THI* element.

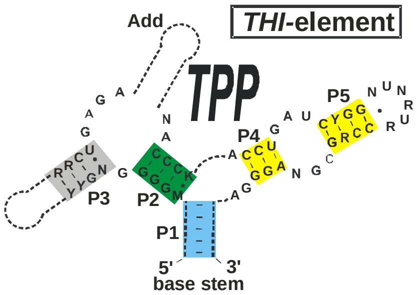

Supplementary Fig. 2. (A) The vitamin B12-specific regulatory RNA elements in δ-proteobacteria. *B12-elements* are in bold with conserved sequence regions in red and conserved helices marked by background sea green, gray, yellow, green, cyan and blue colors. Regulatory hairpins (terminators or sequestors) are marked by background magenta with poly-T regions in blue. Start codons and ribosome-binding sites are in blue and underlined.

GM_CobU
TCAGGTGCTTTTGCCTCATGGCAGAAGGTAAAAGGGAAAAGGGTGCGACTCCCTTGCTGTCCCGCAACTGTGAACGGTGATGAAAGCCGCAACGATGCCACTGATCTGGTTTCCATGATGAA
ACCCCGGGAAGGCGCGGCGAGTAAAGTGATCCGTGAGCCAGGAAACCTGCCTGACGTCAGCTTCACAGGACCTCCGTGGAAGAGGCTCCGAGGCCCGGCGTTCGGCTTTTCACCGGTTTTTG
GACCCCGCTTCCCTTCAGGAGGCGGGGTTTTTCATTTATG **terminator/sequestor**

GS_CobU
GTTCAGGTGCTTCCACTCCCACGGTGGAAGGTAAAAGGGAAAAGGGTGAGAATCCCTTGCTGTCCCGCAACTGTGAACGGTGATGAAAGCCGCAGCGATGCCACTGGAGAGTCCTTGTTGATGTG
AAGGCGTGAAATACGCCTTCTTCCGGGAAGGCGCGGCG
AGTAAAGTGATCCGTGAGCCAGGAAACCTGCCCGAACGCATGAGCAGCGGCCTCCGAGGAAGAGGTGCCGAAGCCATCGTCAATTCATCCACGGTATTCGAGCCCCGCCCCTTGTCAGGAGG
CGGGGTTTTTCATTCATG **terminator/ sequestor**

GS_CbiM
CCTTCGGGCGTAAGGGAAACCGGTGCAATACCGGTGCTGCCCCGCAACTGTGAGGGGGGACCGATGCCGCAGTGATGCCACTGTCCGAAGGGACGGGAAGGCGCGGCAGGCGGGATGATCCC
CGAGCCAGGAGACCTGGCTGGAAGG
CGACAACACACTCCGACCGAGGGGAAGGGAGGTTGTATGGGGAGCAGAGCATATCCACTGCGGCTATGAGGTCGTTCCGGCACGCGAGGGCAAGAACGGCGACGGATCATAACTTCATAGGG
AGCACGCAATGAAACGAA **sequestor?**

DD_CbiK
ATTCAGGTGCCCTGCGGGGCTTGATAGGGAATCCCGTTAAAGTCGGGAGCGGACCCGCCGCCGTAAGTCCACGATACGCCGCATCCACACCGTGCCACTGGGAAAAATTCCCGGGAAGGCCG
GATGCCGCGGGACAAGCCGGAAGACCTGCCTGAGTGTACAGGATGACTTTTTGGGCACGGGGCTTTGCCGCAAAAAGTCCGTCACGGTGACCGGAGCGCCCCTTCCTGCCCATGCAGGAGGC
AGCGCTCTTTATTTTTACCGTGACTATATCAATGCCCCAGATTCTCTGGAGGTTTCCTTCATGAAACTGC **terminator**

DV_CbiK
ACGAGTCAGGTGCCCTGAGCGGCTTGATAGGGAATCCCGTGTGAATCGGGAGCGGACCCGCCGCCGTAAGCCCATAAAAGACCTTCTCCCCCGTGTGCCACTGGACGACCGTCCGGGAAGGC
CGGAGCAGGTCGGGTGAGCCGGAAGACCTGCCTGACACGTCCGATGCCATGCCCGGCGACAACGGGGCTTTTGTCCGACCGGGCCCACGGTGGCGACCCGACACATGCCGCGCCCGGCGCGC
AGCCCTGTGGTCATCCGTCCTGCCGTCCACGCCAATTGCCCGCCAACCTCCCTGGAGGTTGCCATGTCTCGCC **sequestor**

DV_NrdDG
CTCCGGTTTCGGGAATCCGGTGCGAGTCCGGAGCGGTCCCGCCGCTGTCATCCCTGCGCCTCGGGCGCCACGCCACAGCCATGAAGCCACTGCCCCCTGCGGGCGGGAAGGCGGTTGTGAGT
GGGGAGAGCCAGAATACCGGGCCGGAGCAGTCTCGATCCCTCGTTGCACGCGGATGTACCGGGGGTTCAGCCATTCACCATTCGCGGTGCGGGTGGCGACGTTTCCGACGTCGACCCGTGCC
TCCGCACGCAGTGCCGCCCGTTGCCATGACGGGGTCCGCCGGTCTTGTGCGTGCCCGGAGCCAAGCATCCTATGCCCAAGAAGATT **sequestor**

DA_NrdDG
GGATCACGGTGCCCAACGGGAGCTTAAATGTCTCGTGAGGCTTGATAGGGAAGAGGGGTGCAACTCCCCCGCGGACCCGCCGCTGTAAACGTGGACAACAGGCTCCATGCCACTGGTTTCGA
CCGGGAAGGCGCCTGAAGGATGAAGCGTAAGCCAGAAGACCTGCCTGATCCCAACACTCAATGATTCCTCGTGGTAAAGGGAGAGTGACGCATGCATCCGGATTTAAATACGCGAAGTCCG
CAGGGAGCTTTGGCCCCTGCGGACTTTTTTGTGTTTCCGGTGCAGACTGTCTCCTGTGCCGCGGTTTAACCTGATGAAGGAGCAGTCGATGCCAGAGAC **terminator**

DA_BtuBCDF
GTCCACCGGTGCCCGCCAGGGTGAAAAGGGAAGAGGGGTGCAAATCCCCCGCGGACCCGCCACTGTAAGCGAAGACGAAGGGTTGACAGACCACTGGCCGCCTGGCCGGGAAGGTAACCCCG
AGGATGATTCGCGAGCCAGGAGACCTGCCGACATGGACACACCACATTGACCTCCCCGGGAAACAGGGAGAGTGGAGATTAGCGGCGCTTCAAATACGGGAAGCCCGCGCACCTATTTAGGG
CTGCGCGGGCTTTTTTGCGTTGAAGCGGTCCCCTTTTCGCCATTTTCGTTCGGGGCAAAAAGGAGAAATTCGATGAAGAAACA **terminator**

DA_BtuB2
GTTTCAGGTGCTTGTTGCAAGCACAATAGGGAATTCCGTGAAATTCGGGAGTGGGCCCGCCGCTGTAACCAGGGACGATCAACACATTTGGCCACTGACTTAGAGTCGGGAAGGCGTGTTGA
GACGGAAGATCTGGAAGCCAGAAGACCTGCCTGAAACGCAATGGTTGCAACTTTCGTGGCGAAAGTTGAAGCAATAAAGTATCACGGGAGATATGGGATAACCCCGGATCGATGTTTTGTAT
CGGTTCGGGGTTTTTTGCGCTCCGGACACAAACCTGTAGGAGGCTTGTATGAAGAAGA **terminator**

DA_BtuB3
ATTTCAGATGCTCTCACGAGCTTAATAGGGAACCCTGTGTAATTCAGGGACGGGCCCGCCGCTGTAACCGAGGACGAAAGCCGCTTGATGTCACTGCCGAAATTCGGTGGGAAGACGTGGCC
GGTAGGATGATCCGGAAGTCAGAAGACCTGTCTGAAATTTGGTAATGCGCGCTCCTAAGGCAGGGAGACGTATTGAATGTCTTGTGGATAGAAAAGGGATATCCCAGGTCGATGTGTATTCG
GTCTGGGATTTTTTACGCCCGGACCCCAGGAAGATTTTGGGAGGAGCGTACCCCATGAAAACAAT **terminator**
DP_CbiK
TGTTTCAGGTGCTTTGCCTGTGGTAAAAGGTTTAGCTTAATAGGGAATCCTGTGGAATTCAGGAACGGGCCCGCCGCTGTAACTGGGGACAACTTCTGCATTATGTCACTGAACGTTTGTTT
GGGAAGGCGCAGAAGAAGGATGATCCAGAAGTCAGAAGACCTGCCTGCAACAAGTTTTGGTATGCTTCGTGGACAGGAGTGATGCCAGAGGATACGAGGAAAGTTAGGGACTTCCCCGGTCG
ATTTTCGTCCGGAGGGCTCTGTTCTCCCGGACTTTTAGTTGCATCTGTTGGCTGTGTTTGCTGCATCC **sequestor**

(B) Conserved structure of the *B12*-element.

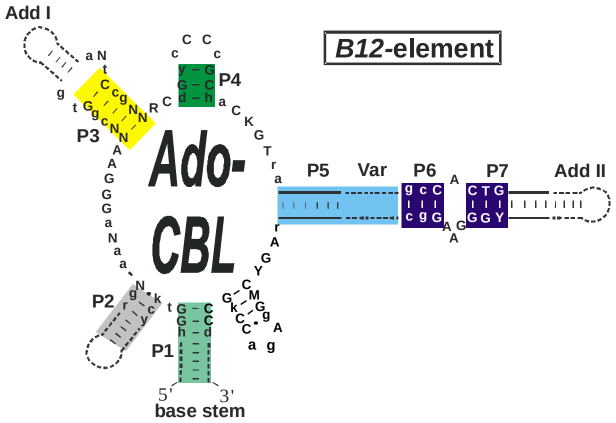

Supplementary Fig. 3. (A) The SAM-specific regulatory RNA elements (S-boxes) in δ-proteobacteria. S-boxes are in bold with conserved sequence regions in red and conserved helices marked by background green, yellow, blue and gray colors. Regulatory hairpins (terminators) are marked by background magenta with poly-T regions in blue. Start codons and ribosome-binding sites are in blue and underlined.

GS_metX
CCTTCTTATCAAGAGTGGTGGAGGGAAAGGCCCTGTGAAACCACAGCAACCGGTCCGGTAGCGGACGCCAGGTGCTAAATCCTGCCCGAAAGGGAGCGATGAGAGGGAGCTTGTGACCACCGACGCGTACA
GGCCCCTTCCCGTTTCCCGGGAGGGGGCCTTTCATTTTCGCCGCCGCGCACGCGCCCGTGGGGAATCATG
GS_METI
CTTATCAAGAGCGACCGAGGGACAGGCCCGGTGACGTCGCGGCAACCTCCCCATGGGGGGAAGGTGCCAATTCCTGCGAGACCGACAGGTTTCGGGAGATAAGGAAGAGCGTGACACCTCACGGTGAATCG
AATCCTCTTCCGCACCCCGGAAGGGGATTTTTCATTGTGGAGGAAACCATG
GM_METI
TTTCTTATCACGAGCGACCGAGGGACTGGCCCTATGACGTCGCGGCAACCCCCCGCAAGGGGACGGTGCCAAATCCTGCGGAACGGCAACGTTCCGGGAGATAAGGAAGAGCGTTGCCGCCGTAACGGCGA
AAAATCCTCTTCCGCACCCCGGAAGGGGATTTTTTCATTCAGGAGGCAAAACCACCATG
GM_METX
TTCTTATCAAGAGTGGTGGAGGGAAAGGCCCTGCGAAGCCACAGCAACCGGTCTTCCGGGTCGCATTCGAGCACGAAGGCGAACCGGAAGGTGACAGGTGCTAAATCCTGCCGGGAGGCAAAGATGAGAAC
GGCGCTTGACGATAGCCACAACTGTCAACAAAGCCCCTTCTCGCCACCGCGGGAAGGGGCTTTTGTTTCCGAACCATTTATTCAGGATTGACGCATG
DA_METX1
CCGTGTTATCACGAGTGGTGGAGGGAATGGCCCTTTGAAACCACAGCAACCGGTCCTGACGTTCAGGATGACAGGTGCTAATTCCACCCCCGCAAGGGGACAGATGAGACGGCGTCTTTGGATACTTGATC
CACCACAGGACGTTTCTTCCTGTTCCACGTATCCACAAGGCCCCATTCTCTCTGGATGGGGCCTTTTTGCGTGGAGAGAAATTTG
DA_METX2
CGTCTTTATCGCGAGTGGTGGAGGGAAAGGCCCTGTGAAGCCACAGCAACCGATCCGCAGATGCGGATGCCAGGTGCTAATTCCTGCCCTGTAACAGGGACAGATGAGGACGGAGCCCGAAAGATACGCGC
TTATCTTTTCCCGTATCGTCAAGGCCCCTCCTGTCACCCTGGGAAGGGGCCTTTTTACGTCAAAGGGTGTCGCGCGTTG
DA_METI
CGTCTTTATCACGAGTGGTGGAGGGACAGGCCCGACGAAGCCACAGCAACCGATCCGCAGGTGCGGATGCCAGGTGCTAAATCCTGCCTGCCGTATGCCATCGCGGATGCGGTCGGAAAGATGAGGACGGT
GCTTCCCGGTTATCCGTCCCTTTGACTGTGCCGGCGAAGCCCCTTCTCATCCCCGAGAGAAGGGGCTTTTTCTATGCCGCCCACAGACCTTGACGAGGGATGACCCATG

(B) Conserved structure of S-box.

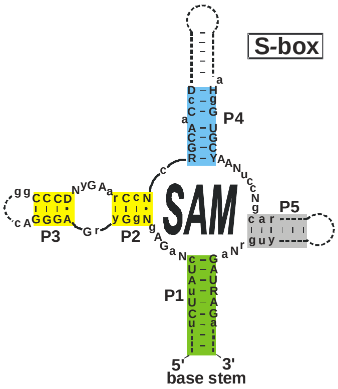

Supplement: Additional data file 1 — Three figures with detailed description of DNA- and RNA-type regulatory sites [file gb-2004-5-11-r90-s1.pdf]
